# NUDT21 Drives T‐Cell Acute Lymphoblastic Leukemia Through Dual Regulation of Alternative Polyadenylation and Transcriptional Activation

**DOI:** 10.1002/advs.202520693

**Published:** 2026-04-02

**Authors:** Conglian Qiu, Jianwei Wang, Zhiheng Li, Qi Ji, Hui Zhang, Qinyi Zhang, Senlin Zhang, Juanjuan Yu, Yanfang Tao, Yijun Wu, Chunxia Shi, Zong Zai, Zimu Zhang, Yizhen Li, Zhenjiang Bai, Shaoyan Hu, Jian Pan, Yang Yang, Shuiyan Wu

**Affiliations:** ^1^ Pediatric Intensive Care Unit Children's Hospital of Soochow University Suzhou China; ^2^ Institute of Pediatric Research Children's Hospital of Soochow University Suzhou China; ^3^ Department of Hematology Children's Hospital of Soochow University Suzhou Jiangsu China; ^4^ St. Jude Children's Research Hospital Memphis Tennessee United States; ^5^ Pediatric Hematology & Oncology Key Laboratory of Higher Education Institutions in Jiangsu Province Suzhou China

**Keywords:** alternative polyadenylation, MYC, NUDT21, T‐ALL, UBE2D3

## Abstract

T‐cell acute lymphoblastic leukemia (T‐ALL) is an aggressive hematological malignancy with limited therapeutic options. Here, we identify the alternative polyadenylation (APA) regulator NUDT21 as a key factor in T‐ALL maintenance through integrated multi‐omics analyses and functional studies. NUDT21 is aberrantly upregulated in T‐ALL patients, and its elevated expression is associated with poor clinical outcomes. Mechanistically, NUDT21 exerts dual oncogenic functions. As a core component of the CFIm complex, NUDT21 promotes distal poly(A) site usage of oncogenic transcripts, most prominently UBE2D3, generating long 3′UTR isoforms with enhanced mRNA stability and increased protein expression. Functionally, UBE2D3 acts as a critical downstream effector that sustains leukemia cell proliferation and survival through MYC‐dependent signaling. Beyond its canonical role in APA regulation, NUDT21 also localizes to transcriptionally active promoters and interacts with lineage‐specific transcription factors, including MYB, RUNX1, and GATA3, to facilitate MYC transcription. Importantly, pharmacological targeting of NUDT21 with ouabain octahydrate induces robust apoptosis in T‐ALL cells by promoting NUDT21 protein degradation and concomitant suppression of UBE2D3 and MYC. Collectively, our findings establish NUDT21 as a multimodal oncogenic regulator and a promising therapeutic target in T‐ALL.

## Graphical abstract

In summary, our study defines a coordinated oncogenic model in which NUDT21 integrates alternative polyadenylation–dependent UBE2D3 stabilization and transcriptional activation to sustain MYC‐driven T‐ALL cell survival, thereby establishing NUDT21 as a central regulatory node and a promising therapeutic target.

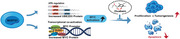

## 1 Introduction

T‐cell acute lymphoblastic leukemia (T‐ALL) is an aggressive hematologic malignancy characterized by uncontrolled proliferation of immature T‐cell progenitors [1, 2, 3]. Despite significant advancements in treatment protocols, approximately 20%–25% of pediatric patients and over 50% of adult patients experience relapse. This underscores the urgent need to identify novel molecular drivers of disease progression [4, 5]. Recent studies have demonstrated that dysregulation of post‐transcriptional gene expression mechanisms—particularly alternative polyadenylation (APA)—plays a crucial role in cancer pathogenesis [6]. APA influences mRNA stability, localization, and translation efficiency by determining the length of the mRNA 3' untranslated region (3' UTR), which is a process critical for oncogene expression and tumor progression [7, 8].

NUDT21, also known as CFIm25, is a pivotal component of the cleavage factor Im (CFIm) complex and serves as a master regulator of APA. It recognizes UGUA motifs to facilitate the selection of poly(A) sites [9, 10]. Notably, large‐scale functional genomics datasets suggest that NUDT21 may act as a pan‐cancer essential gene, highlighting its broad importance in cellular viability across malignancies [11, 12, 13, 14]. However, the context‐specific molecular mechanisms by which it drives oncogenesis in particular cancer types, such as T‐cell acute lymphoblastic leukemia (T‐ALL), remain largely unexplored. This represents a significant gap in our understanding since T‐ALL displays distinct molecular characteristics compared to other leukemias, including unique patterns of oncogene activation and alterations in signaling pathways [1, 15, 16, 17, 18]. Furthermore, emerging evidence indicates that RNA processing factors may extend their functions beyond traditional roles and could be involved in broader gene regulatory networks [7, 13, 19].

The potential multifunctionality of NUDT21 raises several significant biological questions. In this study, we adopt an integrated approach to investigate the role of NUDT21 in T‐cell acute lymphoblastic leukemia (T‐ALL). Through systematic analysis of alternative polyadenylation (APA) patterns, transcriptome profiling, and functional characterization, we aim to elucidate the molecular mechanisms by which NUDT21 contributes to the maintenance and progression of T‐ALL. Our findings may offer new insights into the interplay between post‐transcriptional regulation and leukemia biology while potentially identifying novel therapeutic targets for this aggressive malignancy.

## 2 Results

### 2.1 APA Regulator NUDT21 is Upregulated in T‐ALL and Associated with Poor Prognosis

**FIGURE 1:**
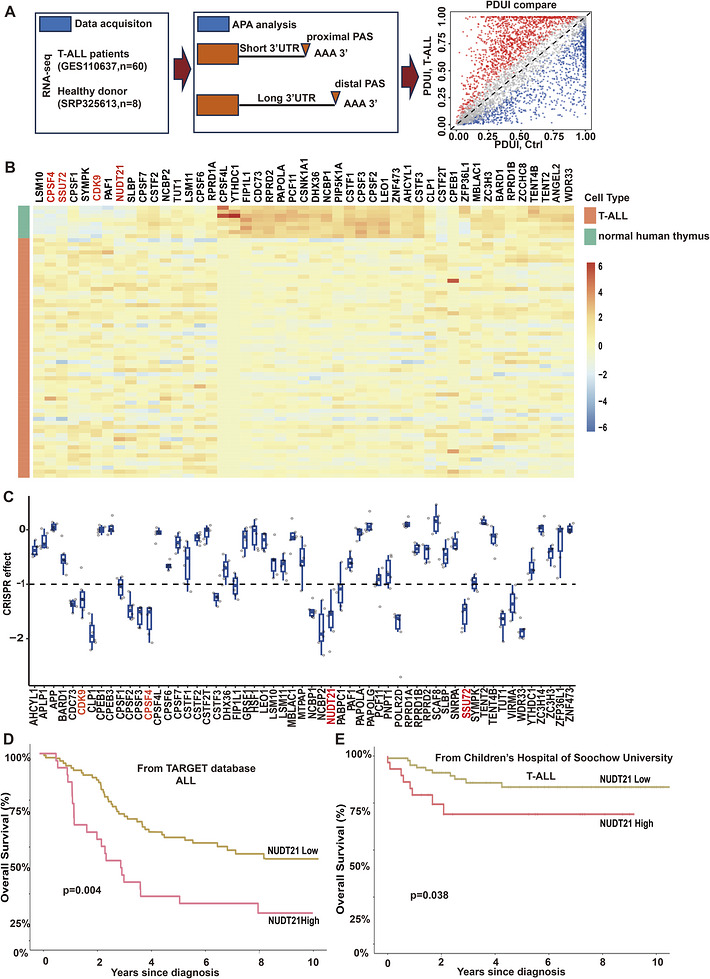
Alternative polyadenylation (APA) dysregulation in T‐ALL biology and prognosis. (A) Computational pipeline for genome‐wide APA analysis in T‐ALL samples. (B) Heatmap of mRNA expression levels of polyadenylation‐related genes across T‐ALL patients‐derived cells (*n* = 60) and normal thymus cells (*n* = 8) based on the datasets of GSE110637 and SRP325613. (C) CRISPR‐mediated effects on polyadenylation‐related genes in T‐ALL cell lines based on data from the DepMap Portal. The dashed line represents an effect value of ‐1. (D) Correlation between NUDT21 expression and overall survival was determined in the TARGET database by Kaplan–Meier analysis. (E) Correlation between NUDT21 expression and overall survival was determined in our cohorts by Kaplan–Meier analysis.

To compare the profiles of alternative polyadenylation (APA) between T‐cell acute lymphoblastic leukemia (T‐ALL) and normal T cells, we collected RNA‐seq data from both T‐ALL patients (GSE110637) and normal T cell samples (SRP325613). Coverage data served as input for the DaPars algorithm. By analyzing the percentage of distal polyA site usage index (PDUI) scores in tumor vs. normal samples, we evaluated the extent of APA‐mediated 3'UTR shortening and lengthening. Our analysis revealed that the majority of genes exhibited APA alterations (Figure 1A). Notably, T‐ALL samples showed more 3'UTR lengthening events (*n* = 1,330) than shortening events (*n* = 1,140) compared to normal thymus (Figure S1A).

We subsequently analyzed the expression profiles of polyadenylation‐related genes associated with GO:0005847 and GO:0031124 in T‐ALL patients compared to normal samples. Our findings revealed that genes such as NUDT21, CPSF4, CDK9, and SSU72 exhibited significantly higher expression levels in T‐ALL patients (Figure 1B; Figure S1B). Following this, we utilized CRISPR screening data from the DepMap portal (https://depmap.org/portal/) to further evaluate the dependency of T‐ALL cell lines on these polyadenylation‐related genes. This analysis indicated that among the upregulated genes in T‐ALL patients, these four demonstrated a high level of dependency (Figure 1C). These results suggest their potential roles as key regulatory factors in T‐ALL. Additionally, we examined another dataset (GSE146901) and found that NUDT21 was significantly elevated in T‐ALL patients when compared to normal controls. In contrast, CDK9, CPSF4, and SSU72 did not show any significant differences between T‐ALL patients and normal controls (Figure S1C). Consistent with our initial observations, NUDT21 was highly expressed across leukemia cell lines (Figure S1D). The prognostic significance of CDK9, CPSF4, NUDT21, and SSU72 in samples from acute lymphoblastic leukemia (ALL) obtained from the TARGET database (https://www.home‐for‐researchers.com/) was further assessed, with a cutoff established at the 75th percentile based on the dataset. Kaplan‐Meier survival curves indicated that ALL patients exhibiting high expression levels of NUDT21 (*n* = 33) experienced significantly shorter overall survival (OS) compared to those with low NUDT21 expression (*n* = 99) (p = 0.004) (Figure 1D). In contrast, high expression of CDK9 and CPSF4 was associated with a favorable prognosis, showing an inverse association with survival outcomes compared to NUDT21. No significant difference in survival was observed for SSU72 expression (Figure S1E). To investigate the clinical relevance of CDK9, CPSF4, NUDT21, and SSU72 specifically in T‐cell ALL (T‐ALL), we conducted transcriptome sequencing on a cohort of 113 pediatric T‐ALL patients at diagnosis. Patients were categorized into high‐expression (*n* = 29) and low‐expression groups (*n* = 84), based on the 75th percentile thresholds for gene expression levels. The baseline characteristics of these patients are detailed in Table 1. Notably, elevated expression levels of NUDT21 were found to be significantly associated with poorer overall survival outcomes (p = 0.038; Figure 1E). In contrast, no prognostic associations were identified for CDK9, CPSF4, or SSU72 expressions—each yielding p‐values greater than 0.2 as shown in Figure S1F. To further validate the independent prognostic value of NUDT21, we conducted a risk factor model analysis for 5‐year overall survival in pediatric T‐ALL patients. High NUDT21 expression was not an independent risk factor in the multivariate analysis, but it remained a significant predictor of poor prognosis in the univariate analysis (Table S1), further emphasizing the prognostic importance of NUDT21 within T‐ALL contexts.

**TABLE 1:** Baseline characteristics of patients with high and low NUDT21 expression.

Variables	High NUDT21 expression (*n* = 29)	Low NUDT21 expression(*n* = 84)	*P* value
Age at diagnosis, median, years			0.315
≥10	9	26	
<10	20	58	
Sex			0.079
Male	20	68	
Female	9	16	
WBC at diagnosis, median (range), ×10^9^/L	149.77 (2.62–520.00)	136.69 (25.68–539.76)	0.192
Hemoglobin at diagnosis, median (range), g/L	99.00 (67.00–155.00)	100.43 (1.05–750.20)	0.324
PLT at diagnosis, median (range), ×10^9^/L	76.00(20.00–238.00)	97.50 (37.00–159.00)	0.924
BM blasts (%)	88.00 (41.50–98.00)	86.00 (22.00–100.00)	0.392
Immunophenotype			1
ETP	1	4	
Non‐ETP	28	80	
NOTCH1/FBXW7 status	12	46	0.45
Fusion gene, n (%)			
SIL‐TAL1	11	17	0.817
MLL rearrangement	0	3	1
HOX11 rearrangement	1	6	1
CNSL			1
Yes	1	2	
No	28	82	
Risk stratification			1
Intermediate risk	12	35	

Abbreviations: WBC, white blood cell; ETP, early thymic precursor; HSCT, hematopoietic stem cell transplantation

### 2.2 NUDT21 Depletion Impairs Leukemia Maintenance Through Suppressing Proliferation and Inducing Apoptosis In Vitro and In Vivo

**FIGURE 2:**
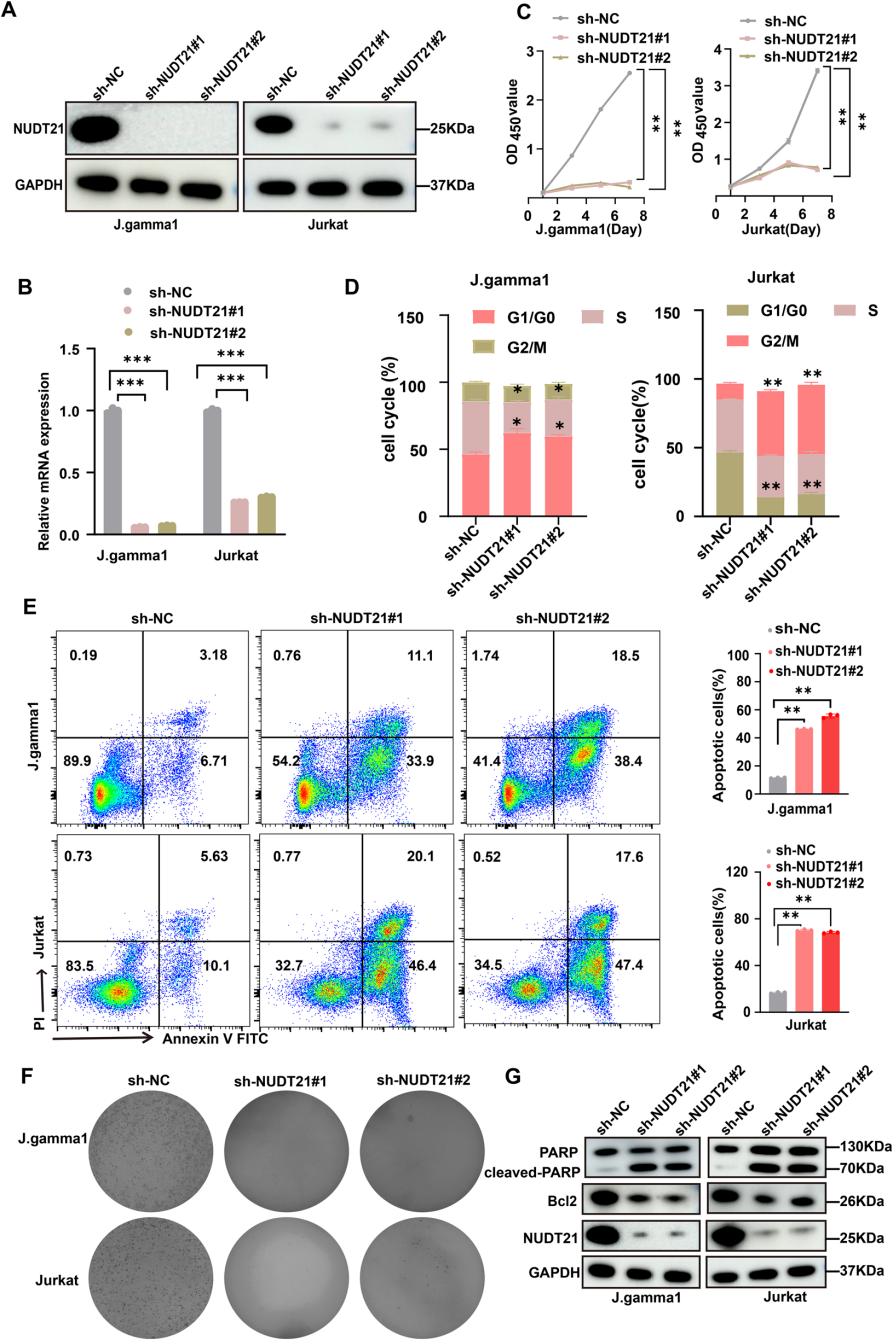
Function of NUDT21 in T‐ALL in vitro. (A,B) NUDT21 knockdown efficiency in J.gamma1 and Jurkat cells was confirmed by Western blotting and qRT‐PCR. (C) Proliferation kinetics assessed by CCK‐8 assay in control vs. NUDT21‐deficient cells. (D) Cell cycle profiling via flow cytometry following NUDT21 depletion. (E) Apoptosis rates measured by Annexin V/PI staining in knockdown models. (F) Colony formation capacity of NUDT21‐silenced cells in soft agar assays. (G) Apoptotic related proteins were increased in the NUDT21‐knockdown cells. Data represent mean ± SD of three biological replicates (^*^ *p* < 0.05, ^**^ *p* < 0.01, ^***^ *p* < 0.001).

We further investigate the functional role of NUDT21 in T‐cell acute lymphoblastic leukemia (T‐ALL). Firstly, we successfully established NUDT21‐knockdown cell lines in two T‐ALL‐derived cell lines, J.gamma1 and Jurkat, utilizing shRNA‐mediated silencing (Figure 2A,B). CCK‐8 proliferation assays demonstrated that the knockdown of NUDT21 significantly inhibited the proliferation of both J.gamma1 and Jurkat cells (Figure 2C). Cell cycle analysis revealed that depletion of NUDT21 resulted in dysregulation of the cell cycle (Figure 2D; Figure S2A). Flow cytometry further indicated a substantial increase in apoptosis following NUDT21 knockdown (Figure 2E). Soft agar colony formation assays showed that silencing NUDT21 in T‐ALL cells led to a reduction in colony numbers and an increase in apoptosis compared to control cells (Figure 2F). Additionally, decreased levels of NUDT21 were associated with elevated expression of the apoptosis‐inducing protein cleaved‐PARP and reduced levels of the anti‐apoptotic protein Bcl‐2 (Figure 2G). Collectively, these findings underscore the critical role of NUDT21 in promoting survival and proliferation within T‐ALL cells, supporting its function in leukemia maintenance.

**FIGURE 3:**
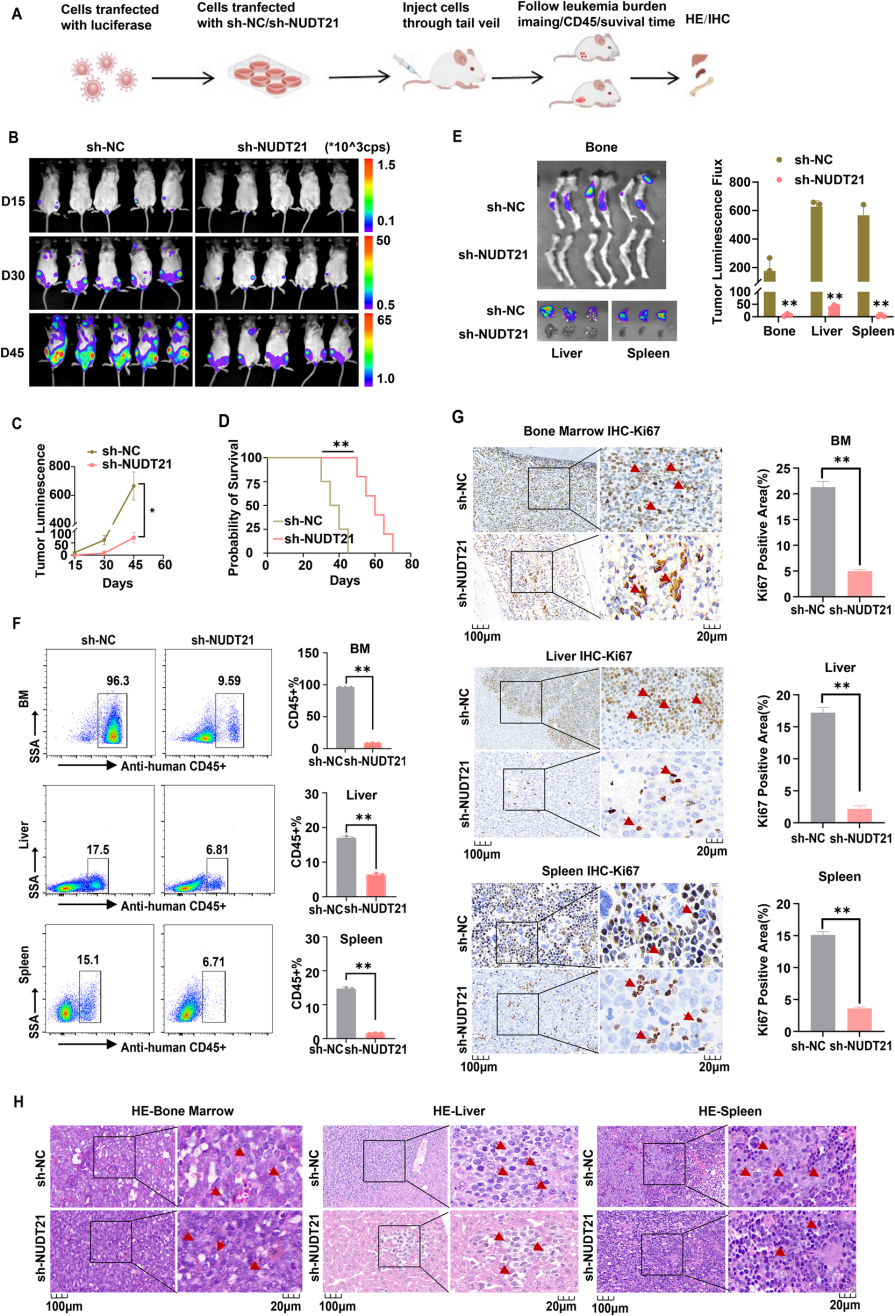
Function of NUDT21 in T‐ALL in vivo. (A) Experimental timeline for T‐ALL xenograft studies. (B) Luciferase intensity was evaluated on the day 15, 30, and 45 in the NUDT21 knockdown group and the control group. (C) The comparison of luciferase intensity between the control group and the NUDT21 knockdown group. (D) Survival curves of mice in both groups (*n* = 5). (E) Demonstration and comparative analysis of luciferase intensity in bone marrow, liver, and spleen between the control group and the NUDT21 knockdown group. (F) Flow cytometric analysis of human CD45^+^ cell frequency in bone marrow, liver, and spleen between the control and NUDT21 knockdown groups. (G) Bone marrow, liver, and spleen were stained with Ki67 antibodies. (H) Representative H&E staining showing reduced leukemic infiltration in liver/spleen/bone marrow.

To further validate the role of NUDT21 in leukemia maintenance, we intravenously injected NSG mice with Luciferase‐labeled T‐ALL cells (either NUDT21‐knockdown or control cells) via the tail vein (Figure 3A; Figure S2B). The leukemia burden was monitored using in vivo bioluminescence imaging. Compared to the control group, NUDT21 knockdown resulted in a significant reduction in the expansion of J.gamma1 cells in vivo (Figure 3B), with markedly lower bioluminescence signals observed in the NUDT21‐knockdown group (Figure 3C). Survival analysis revealed that mice injected with NUDT21‐knockdown cells exhibited significantly prolonged lifespans (Figure 3D). Furthermore, the leukemia burden in the liver, spleen, and bone marrow was substantially reduced in mice engrafted with NUDT21‐knockdown cells compared to controls (Figure 3E). Flow cytometry analysis demonstrated a significant decrease in the proportion of leukemia cells, as indicated by a reduced percentage of human CD45+ cells within the NUDT21‐knockdown group (Figure 3F). Immunohistochemical assessment showed diminished Ki67‐positive proliferating cells present within the NUDT21‐knockdown group (Figure 3G). Additionally, hematoxylin and eosin (H&E) staining of liver and spleen tissues confirmed reduced infiltration of J.gamma1 leukemic cells from the NUDT21‐knockdown cohort into these organs (Figure 3H). In summary, these findings demonstrate that deficiency of NUDT21 significantly impedes leukemia progression in vivo, underscoring its critical role in T‐ALL maintenance.

### 2.3 NUDT21‐Driven UBE2D3 Distal Polyadenylation Enhances T‐ALL Oncogenicity

**FIGURE 4:**
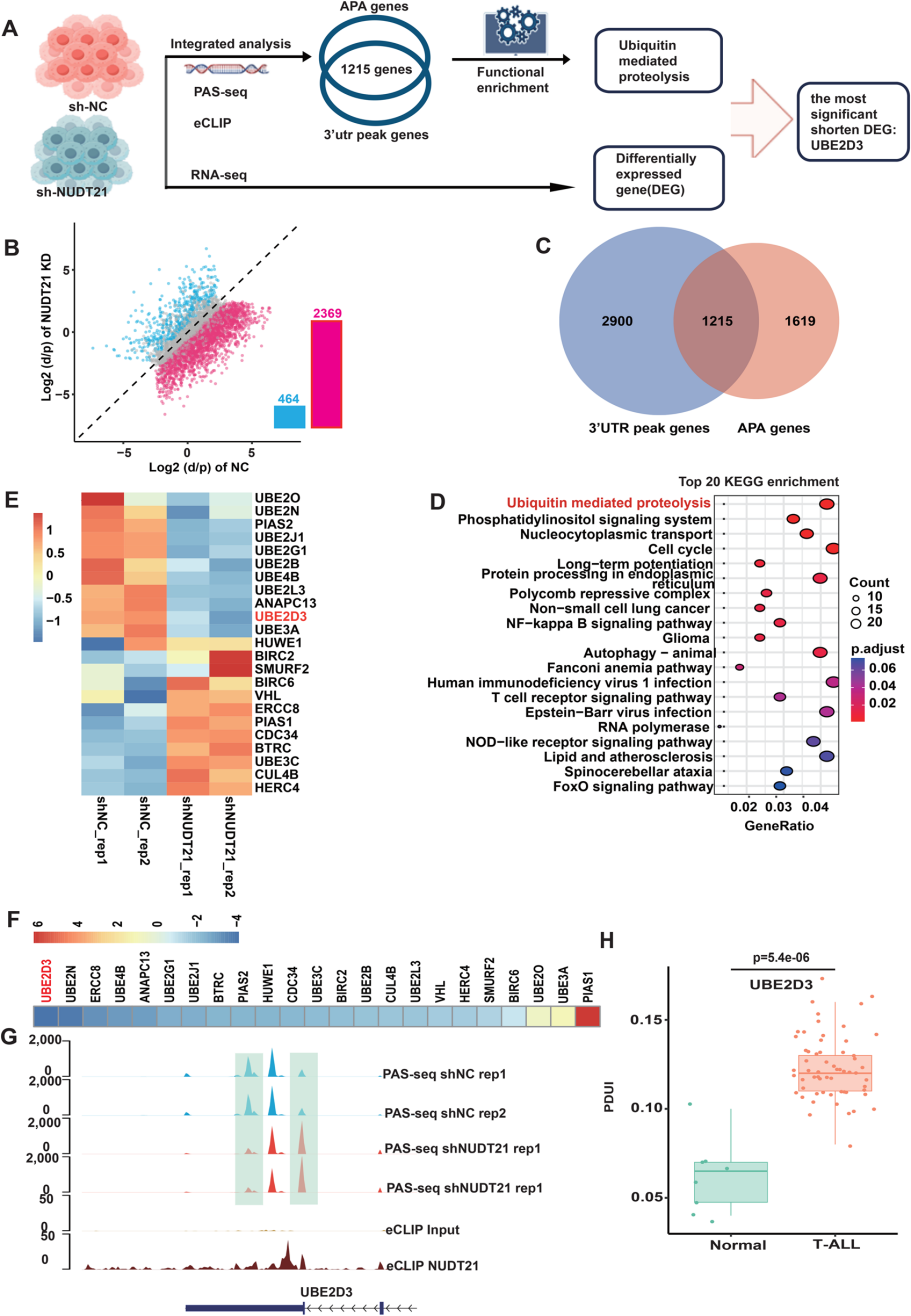
NUDT21 promotes alternative polyadenylation selection of UBE2D3 in T‐ALL cells. (A) NUDT21 mechanistic pathway exploration diagram on APA regulation. (B) Dot plot showing expression ratios of distal and proximal polyA sites between NUDT21 knockdown and NC. Each point represents a gene. Red points represent genes shifted to proximal polyA sites after NUDT21 knockdown. Blue points represent genes shifted to distal polyA sites after NUDT21 knockdown. Gene numbers are indicated in the figure. “d” indicates the expression of the isoform using the distal polyA site. “p” indicates the expression of the isoform using the proximal polyA site. (C) Venn diagram showing the intersection of genes with APA events and genes containing eCLIP‐seq peaks in the 3’UTR region. Gene numbers are indicated. (D) KEGG analysis of the overlapping genes in C. The most significant pathway is highlighted in red. (E) Heatmap showing the expression of genes in ubiquitin mediated proteolysis pathway. (F) Heatmap showing the relative expression difference (RED) of genes in ubiquitin mediated proteolysis pathway. (G) Genome browser plots showing the PAS‐seq and eCLIP‐seq signals around the UBE2D3 3’UTR locus. (H) Comparative analysis of PDUI scores of UBE2D3 between T‐ALL and normal samples.

To investigate the mechanistic role of NUDT21 in T‐ALL, we employed an integrated multi‐omics strategy, as outlined in Figure 4A. As a core subunit of the CFIm complex that mediates alternative polyadenylation (APA), NUDT21 plays a critical role in regulating 3'‐end processing and polyA site selection during mRNA maturation. To determine whether NUDT21 functions through its canonical role in poly A site selection, we performed PAS‐seq in J‐gammal cells following NUDT21 knockdown, which enables precise quantification of polyA site usage. Read distribution analysis showed that most reads were localized in 3'UTR regions (Figure S3A,B) and significantly enriched upstream of transcriptional termination sites (TTS) (Figure S3C,D), which confirms the reliability of the data. Notably, NUDT21 depletion induced APA alterations in 2,833 genes, with 2,369 genes showing increased proximal poly A site usage and 464 genes exhibiting preferential distal site selection compared to controls (Figure 4B; Table S6).

To further explore the rules of NUDT21‐mediated poly(A) site switching, we performed sequence feature analysis on the flanking regions of poly(A) sites in genes with 3'UTR shortening or lengthening upon NUDT21 knockdown. We first quantified the distribution of the UGUA motif, a known NUDT21 binding motif, upstream of both proximal and distal poly(A) sites. For genes undergoing shortening, the UGUA motif was significantly enriched in distal regions compared to proximal regions; conversely, the opposite pattern was observed for lengthening genes (Figure S4A). Nucleotide distribution analysis revealed that shortening genes displayed a U‐rich distal region, while lengthening genes harbored a U‐rich proximal region, consistent with UGUA motif distribution (Figure S4B). Furthermore, we also compared the PAS motif frequency between the two groups of genes. As a result, the canonical and strongest PAS motif–AAUAAA distribution aligned with the patterns of UGUA and U‐rich features for shortening genes, while lengthening genes showed comparable proximal/distal AAUAAA (Figure S4C). These results indicated that NUDT21 may modulate canonical PAS selection via a “synergistic compatibility‐driven functional binding” model, integrating UGUA recognition, U‐rich environment preference, and AAUAAA distribution.

To determine whether NUDT21 directly regulates 3'‐end processing of these genes, we performed NUDT21 eCLIP‐seq. A total of 20,499 peaks were identified from the eCLIP‐seq data, of which 42% of the peaks localized to 3'UTRs (Figure S5A and Table S7). Consistent with previous reports [9, 10], NUDT21 was found to bind specifically to the UGUA motif, which was present in approximately 56% of the identified peaks (Figure S5B). Among the genes exhibiting APA events, 1,215 (≈43%) showed NUDT21 binding in their 3'UTRs (Figure 4C; Table S8). KEGG pathway analysis of these 1215 genes revealed the most significant enrichment in the ubiquitin‐mediated proteolysis pathway (Figure 4D; Table S9). To examine the differential expression of APA‐affected genes, we conducted RNA‐seq on samples with NUDT21 knockdown and negative control counterparts, followed by differential expression analysis (Fold Change > 1.2, FDR < 0.01) (Figure S6A and Table S10). This analysis identified 869 downregulated and 709 upregulated genes among the APA‐affected genes (Figure S6B and Table S11). Notably, 42%–43% of genes in all expression categories (upregulated, downregulated, or unchanged) exhibited NUDT21 binding in their 3'UTRs (Figure S6C). Further GO enrichment analysis of the downregulated genes with NUDT21 binding in their 3'UTRs revealed significant enrichment in biological processes, including regulation of chromosome organization, chromosome segregation, and nuclear chromosome segregation (Figure S6D), while the upregulated genes showed predominant enrichment in vesicle‐mediated transport to the plasma membrane and protein polyubiquitination (Figure S6E).

In a further step, we compared the expression of the genes involved in ubiquitin‐mediated proteolysis between NUDT21 knockdown groups and the negative controls (Figure 4E), as well as their relative expression difference (RED) in distal poly A site usage (Figure 4F). We ultimately focused on the UBE2D3 gene, as it was the most significant 3’UTR shortening and downregulated gene. Integrated visualization of PAS‐seq and eCLIP‐seq signals for UBE2D3, UBE2N, and UBE2J1 was shown (Figure 4G; Figure S6F). We also performed KEGG pathway analysis on genes with APA events in T‐ALL patients compared to normal samples, and the results showed that the ubiquitin‐mediated proteolysis pathway was likewise significantly enriched (Figure S7A). Specifically, T‐ALL samples predominantly expressed UBE2D3 transcripts utilizing distal poly A sites (Figure 4H; Figure S7B), consistent with the results of the cell line experiments.

### 2.4 UBE2D3 Acts as a Critical Downstream Effector of NUDT21 to Support Proliferation and Suppress Apoptosis in T‐ALL

**FIGURE 5:**
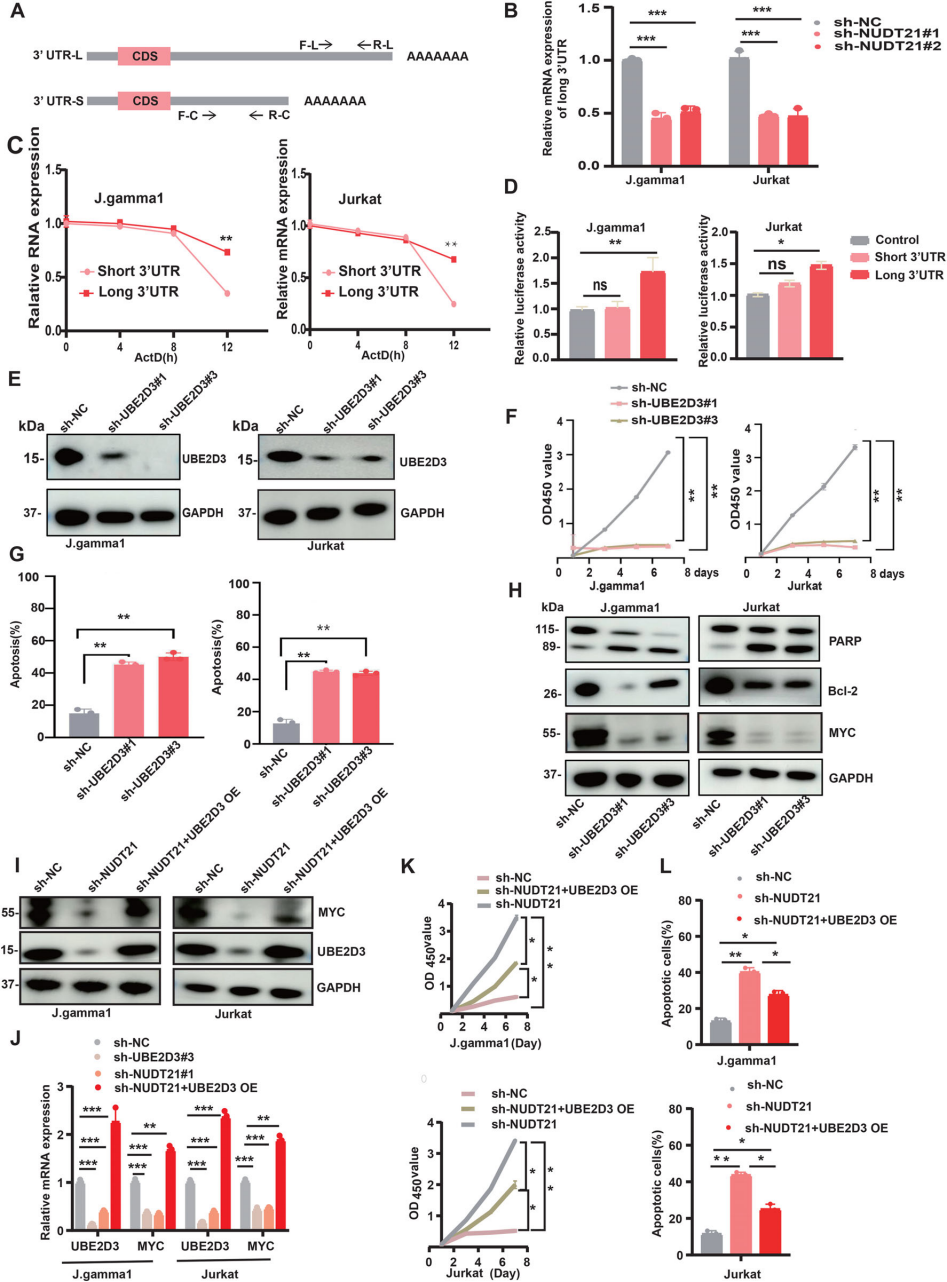
Role of UBE2D3 3’UTR length‐dependent regulation in T‐ALL. (A) Specific PCR primers were designed to quantify the expression of UBE2D3 long and short 3'UTR isoforms. (B) qRT‐PCR was applied to detect the UBE2D3 long 3’UTR after sh‐NUDT21 transfection. (C) UBE2D3 mRNA mRNA stability in T‐ALL cells treated with actinomycin D (5 µg/ml) was determined by qRT‐PCR. (D) The effect of long or short 3’UTR UBE2D3 on protein activity was measured by luciferase reporter assay. (E) Western blot of UBE2D3 protein levels in J.gamma1 (left) and Jurkat (right) cells transfected with the UBE2D3 knockdown plasmids. (F) Proliferation of J.gamma1 (left) and Jurkat (right) cells was examined by CCK8 assay. (G) Apoptosis detected by flow cytometry of J.gamma1 (top) and Jurkat (bottom) cells transfected with UBE2D3 knockdown plasmids. (H) The level of apoptotic protein and MYC was detected in J. gamma1 (left) and Jurkat (right) cells transfected with UBE2D3 knockdown plasmids. (I) Western blot analysis showing MYC protein levels upon UBE2D3 knockdown and upon UBE2D3 re‐expression in NUDT21‐knockout cells. (J) UBE2D3 and MYC mRNA expression were verified by RT‐PCR upon UBE2D3 knockdown and upon UBE2D3 re‐expression in NUDT21‐knockout cells. (K) Cell proliferation assay and (L) apoptosis analysis in control, NUDT21‐knockout, and NUDT21‐knockout cells with UBE2D3 re‐expression.

We designed specific PCR primers to quantify the expression of UBE2D3 long and short 3'UTR isoforms (Figure 5A). As shown in Figure 5B, knockdown NUDT21 reduced the long‐3’UTR isoform of UBE2D3. We also found that UBE2D3 mRNAs with long 3’UTRs were more stable than those with short 3’UTRs (Figure 5C). To examine the effect of the long and short 3’UTR isoforms of UBE2D3 on its protein production, we employed a dual‐luciferase reporter system, which demonstrated significantly higher luciferase activity from long 3'UTR constructs compared to short 3'UTR variants (Figure 5D), which was consistent with the increased mRNA stability conferred by the long 3'UTR leading to higher protein yield. To investigate the biological significance of UBE2D3 in T‐ALL biology, we performed knockdown experiments in T‐ALL cells. Compared to control vector‐transfected cells, UBE2D3 knockdown (Figure 5E) resulted in significant growth inhibition and increased apoptosis (Figure 5F,G). Consistent with these phenotypic changes, we observed upregulation of the apoptotic marker PARP and downregulation of the anti‐apoptotic protein BCL‐2 and MYC (Figure 5H). These findings demonstrate that the oncogenic function of NUDT21 in T‐ALL is mediated through UBE2D3‐dependent mechanisms. To establish the causal relationship between UBE2D3 and NUDT21‐dependent cell survival, we performed rescue experiments in NUDT21‐knockout T‐ALL cells. Notably, ectopic expression of UBE2D3 not only restored UBE2D3 protein levels but also concomitantly increased MYC expression (Figure 5I,J). Functionally, re‐expression of UBE2D3 significantly rescued the proliferation defects and attenuated apoptosis induced by NUDT21 loss (Figure 5K,L). These results demonstrate that UBE2D3 functions as a critical downstream effector of NUDT21, and that MYC operates as an essential component within the NUDT21‐UBE2D3 signaling axis to sustain T‐ALL cell survival.

### 2.5 NUDT21 Sustains Tumor Cell Survival Through Direct Transcriptional Activation of MYC

Given that the cleavage and polyadenylation process has been reported to be associated with transcription initiation [20, 21], we hypothesized that NUDT21 might exert its oncogenic functions in T‐ALL through both APA‐mediated mRNA processing and direct transcriptional regulation. To investigate this possibility, we performed NUDT21 Cut &Tag sequencing, which demonstrated that approximately 45% of the binding peaks were localized to promoter regions (Figure S8A and Table S12), indicating potential transcriptional regulatory activity.

**FIGURE 6:**
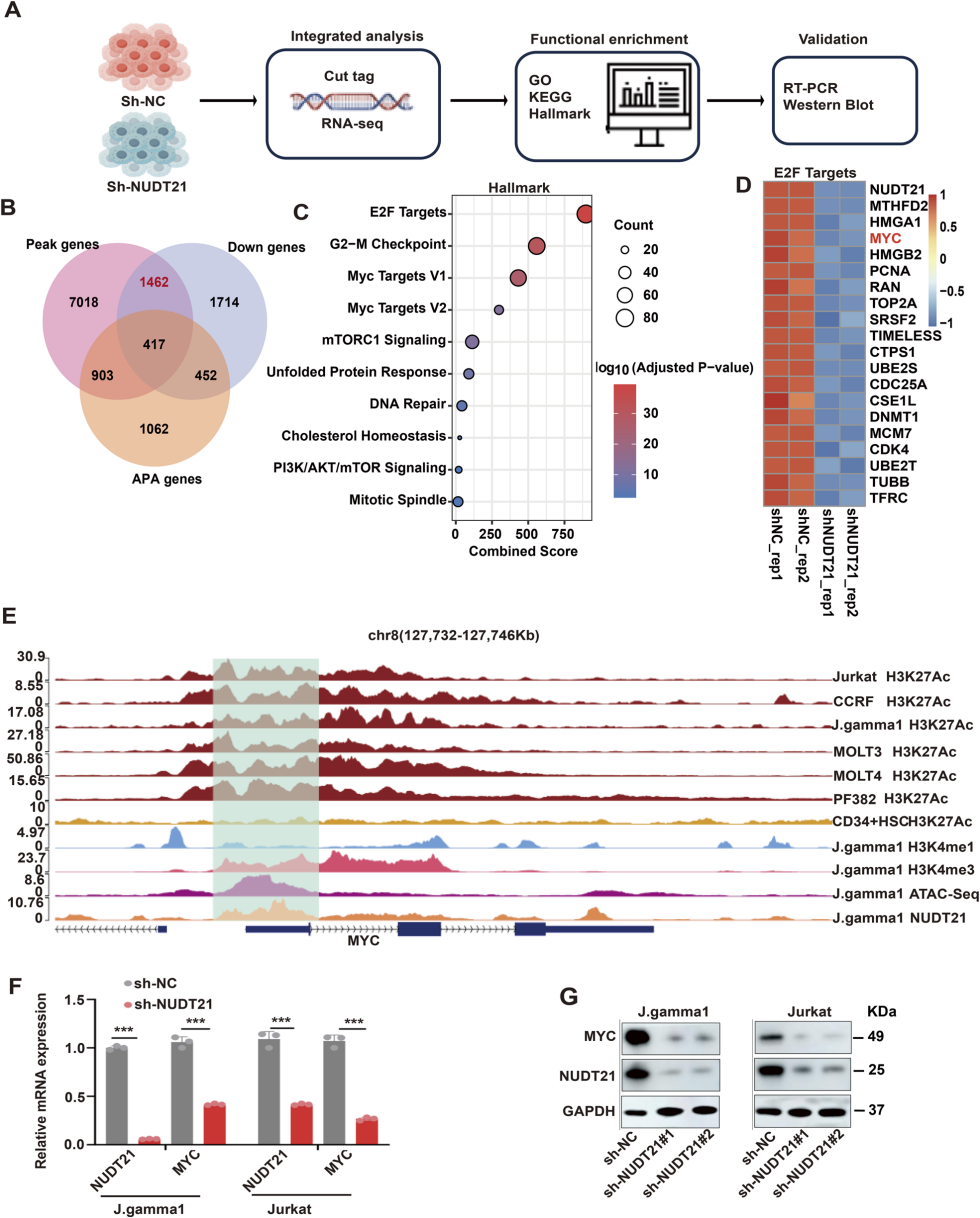
Integrated genomic profiling reveals NUDT21‐mediated transcriptional co‐regulation. (A) NUDT21 mechanistic pathway exploration diagram on trans‐regulation. (B) Venn diagram showing the intersection among genes with APA events, downregulated genes, and genes containing Cut &Tag peaks. The number of downregulated genes with Cut &Tag peaks but without APA events is highlighted in red. (C) Bubble plot showing the top 10 enriched HALLMARK pathways of the 1462 genes in B. (D) Heatmap showing the top 20 downregulated genes in the dataset of E2F targets. (E) Signals of H3K27ac/H3K4me3/H3K4me1 ChIP‐seq across cell lines, ATAC‐seq in J.gamma1, and NUDT21 Cut &Tag in J.gamma1 at the MYC promoter. (F) mRNA expression of MYC was downregulated following NUDT21 knockdown in T‐ALL cells (J.gamma1 and Jurkat). (G) MYC protein level was decreased following NUDT21 knockdown in T‐ALL cells.

Through integrated analysis of NUDT21 Cut &Tag, RNA‐seq, and PAS‐seq datasets (Figure 6A), we identified 1,462 downregulated genes that exhibited NUDT21 binding but showed no detectable APA alterations (Figure 6B; Table S13), suggesting their regulation occurs primarily at the transcriptional level. We identified significant enrichment in several critical biological processes, including DNA replication checkpoint signaling, RNA processing and splicing via transesterification reactions with bulged adenosine as nucleophile (Figure S8B and Table S14), as well as pathways related to cell cycle regulation, DNA replication, and spliceosome function (Figure S8C and Table S15).

Hallmark pathway analysis of these transcriptionally regulated genes revealed significant downregulation in several oncogenic pathways, including E2F Targets, G2M Checkpoint, MYC Targets V1, and MYC Targets V2 (Figure 6C; Table S16). Examination of the top 20 downregulated genes in these pathways (Figure 6D; Figure S8D–F) consistently identified key regulators such as HMGA1, CDK1, HMGB2, MYC, and PCNA. The recurrent involvement of MYC across all analyzed pathways strongly suggests that NUDT21 promotes T‐ALL maintenance not only through its canonical role in APA regulation but also via transcriptional activation of MYC.

To further validate the transcriptional regulatory function of NUDT21 beyond APA regulation, we integrated multi‐omics datasets: (1) NUDT21 Cut &Tag data; (2) H3K27ac ChIP‐seq data (generated across multiple T‐ALL cell lines, including Jurkat, CCRF, J.gamma1, MOLT3, MOLT4, and PF382, as well as CD4+ hematopoietic stem cells [HSCs]); (3) H3K4me1 and H3K4me3 Cut &Tag data (from J.gamma1 cells); and (4) chromatin accessibility profiles (ATAC‐seq). This integrative analysis exhibited NUDT21 specifically binds to the chromatin‐open and transcriptionally active promoter regions of oncogenes such as MYC (Figure 6E), HMGA1, CDK1, and HMGB2 (Figure S9), suggesting that it may function as a transcriptional co‐activator and enhances oncogene transcription by targeting these active promoters in T‐ALL. To functionally validate these findings, we performed qRT‐PCR and Western blot analyses following NUDT21 knockdown. These experiments demonstrated significant downregulation of MYC expression at both mRNA and protein levels (Figure 6F,G), providing direct evidence for NUDT21‐mediated transcriptional regulation of this critical oncogene.

### 2.6 NUDT21 Cooperates with Master Transcription Factors to Promote MYC Transcription

**FIGURE 7:**
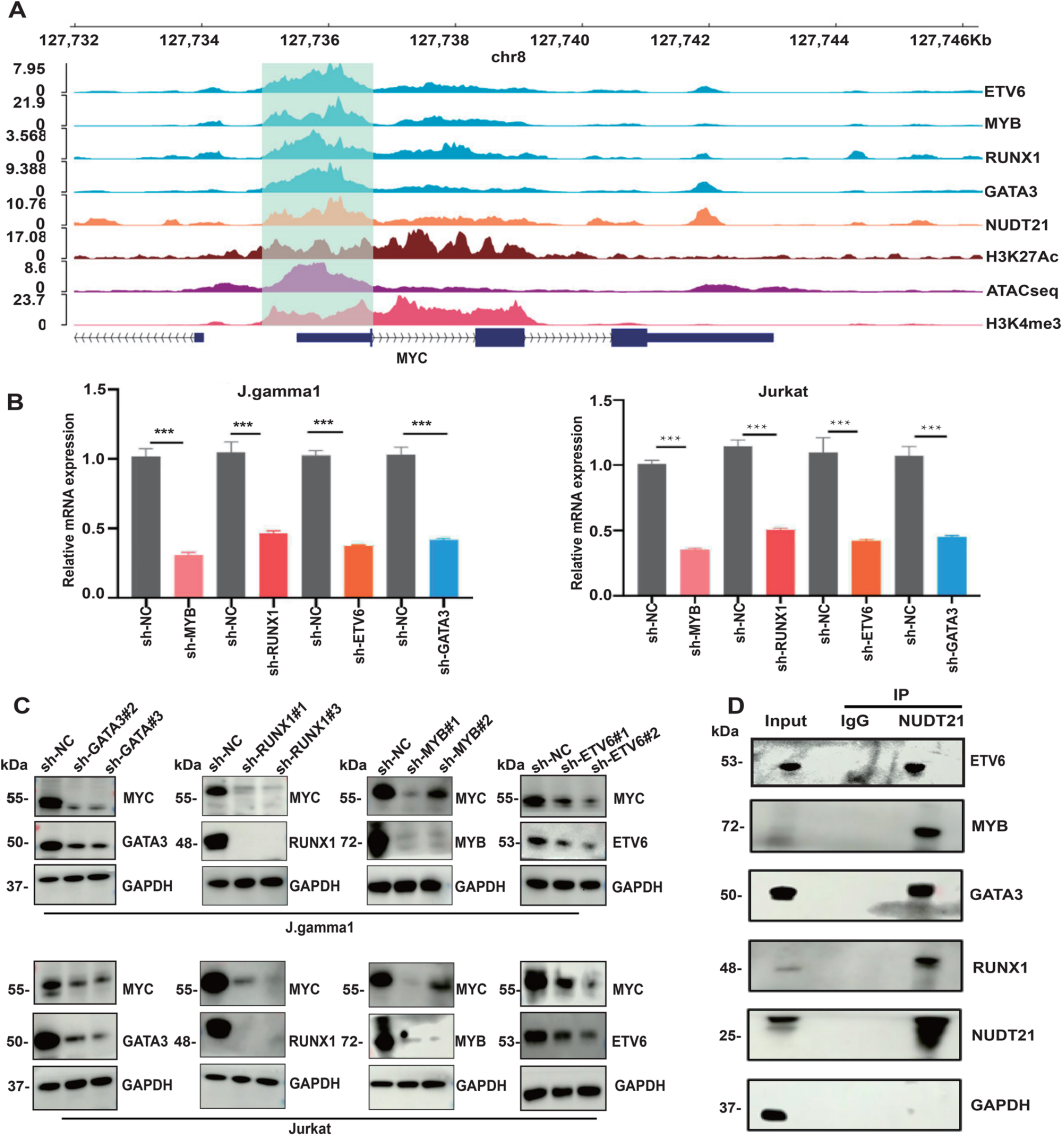
Integrated analysis of NUDT21 co‐regulatory network. (A) Genome browser showing the Cut &Tag signals of NUDT21, ETV6, RUNX1, GATA3, and MYB at the MYC gene locus. The region near the promoter is highlighted in the figure. (B) qRT‐PCR assays demonstrate that knockdown of any individual gene (ETV6, RUNX1, GATA3, or MYB) significantly downregulates MYC expression. (C) MYC protein level was reduced after knockdown of RUNX1, GATA3, MYB, or ETV6. (D) Co‐immunoprecipitation (Co‐IP) experiments confirm physical interactions between NUDT21 and ETV6, RUNX1, GATA3, or MYB.

Building upon our previous findings that identified multiple master transcription factors (TFs) associated with T‐ALL survival [22, 23], we sought to delineate their functional relationships with the NUDT21‐MYC axis. We integrated Cut &Tag signals of ETV6, MYB, RUNX1, and GATA3 with those of NUDT21, and identified substantial overlap in their binding peaks (Figure S10A,B). To test whether NUDT21 recruits other lineage‐specific transcription factors (TFs) to the *MYC* promoter, we performed ChIP‐qPCR for ETV6, RUNX1, MYB, and GATA3 following NUDT21 knockdown. NUDT21 depletion did not reduce binding of these TFs to the MYC promoter (Figure S10C), suggesting that NUDT21 likely functions downstream of or in parallel to TF‐DNA binding, possibly by modulating the activity or stability of the transcriptional complex through protein‐protein interactions. This overlap was evident at the promoter region of MYC (Figure 7A), as well as the promoter regions of HMGA1, CDK1, and HMGB2 (Figure S11), suggesting their potential cooperative regulation of transcription for these oncogenes. Functional interrogation through targeted knockdown experiments demonstrated that individual depletion of each TF (ETV6, MYB, RUNX1, or GATA3) significantly reduced MYC mRNA and protein levels (Figure 7B,C). Co‐immunoprecipitation assays further identified physical interactions between NUDT21 and ETV6, MYB, GATA3, or RUNX1 (Figure 7D). These results implied that NUDT21 contributed to the integrity of this TF‐based regulatory unit for MYC transcription.

### 2.7 Ouabain Octahydrate Triggers Apoptosis in T‐ALL Through NUDT21 Degradation and Consequent UBE2D3/MYC Suppression

**FIGURE 8:**
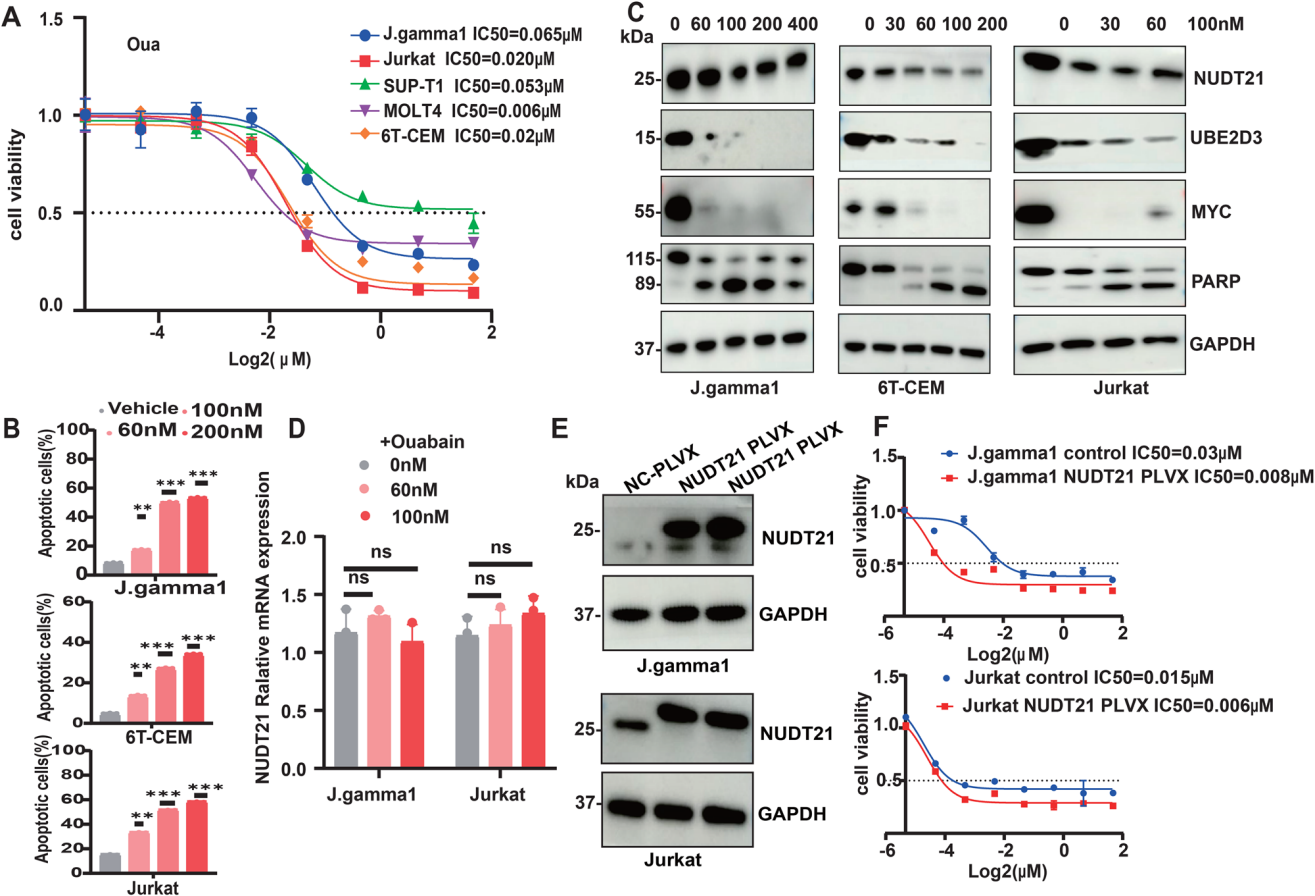
Ouabain octahydrate (Oua) exhibits significant anti‐tumor effects in T‐ALL through NUDT21 inhibition. (A) Cell viability of T‐ALL cell lines with Oua treatment; (B) Cell apoptosis of T‐ALL cells (J.gamma1, 6T‐CEM, Jurkat) treated with different concentrations of ouabain octahydrate (Oua). (C) Western blotting confirmed a decrease of NUDT21, MYC, and UBE2D3, and elevated levels of apoptosis‐related proteins (cleaved‐PARP and Bcl‐2), upon drug treatment. (D) NUDT21 mRNA expression was verified by RT‐PCR after treatment with Oua. (E) Western blotting confirmed the NUDT21 level in these NUDT21‐overexpressing cells. (F) Cell viability was assessed in these NUDT21‐overexpressing cells after treatment with Oua.

A previous report [24] indicated that ouabain octahydrate (Oua) suppresses NUDT21 protein expression in prostate cancer. Based on this observation, we treated T‐ALL cell lines with Oua in vitro for 24 h and performed cell viability assays to determine IC_50_ values. Notably, all five tested T‐ALL cell lines exhibited marked sensitivity to Oua treatment (Figure 8A). The more sensitive Jurkat, 6T‐CEM, and J.gamma1 cell lines were therefore selected for subsequent analyses. Flow cytometric analysis revealed a dose‐dependent increase in apoptotic T‐ALL cells following Oua treatment (Figure 8B). Consistently, exposure to Oua at increasing concentrations for 24 h resulted in a pronounced reduction in the target protein NUDT21. Western blot analysis further confirmed elevated levels of apoptosis‐associated markers, including cleaved PARP and Bcl‐2, while the proliferation‐related protein MYC was markedly downregulated. In addition, Oua treatment reduced the protein expression of UBE2D3 and MYC concomitantly with NUDT21 suppression (Figure 8C). To determine whether the anti‐leukemic effects of Oua are mediated through NUDT21 degradation, we performed rescue experiments by ectopic overexpression of NUDT21 in T‐ALL cells. Although Oua treatment significantly reduced endogenous NUDT21 protein levels without affecting its mRNA expression (Figure 8C,D), enforced expression of NUDT21 (Figure 8E) failed to rescue the decrease in cell viability induced by Oua treatment (Figure 8F). Notably, co‐treatment with the proteasome inhibitor MG132 effectively prevented Oua‐induced NUDT21 protein reduction (Figure S12), indicating that Oua promotes NUDT21 degradation in a proteasome‐dependent manner. Collectively, these results demonstrate that Oua exerts potent anti‐tumor activity in T‐ALL cells in vitro primarily by inducing proteasomal degradation of NUDT21, leading to suppression of the UBE2D3/MYC axis and activation of apoptotic signaling.

## 3 Discussion

Our study provides compelling evidence that NUDT21 serves as a critical oncogenic regulator in T‐ALL through its dual functions in both post‐transcriptional regulation and transcriptional control. The identification of widespread 3′UTR alterations in T‐ALL reinforces accumulating evidence that alternative polyadenylation (APA) dysregulation represents a hallmark of cancer pathogenesis [8, 25, 26]. Importantly, among multiple polyadenylation‐related factors, NUDT21 emerged as the predominant APA regulator in T‐ALL, with its overexpression strongly correlating with poor clinical outcomes across independent cohorts. This finding extends recent work demonstrating the importance of APA factors in hematologic malignancies [7, 11, 14, 27], while providing T‐ALL‐specific insights.

The mechanistic characterization of NUDT21 revealed its essential role in maintaining the malignant phenotype through multiple pathways. Our demonstration that NUDT21 depletion induces cell cycle arrest, apoptosis in vitro, and markedly impaired leukemia progression in vivo, providing functional validation of its oncogenic role. These findings align with emerging concepts about the importance of RNA processing in cancer cell survival [28, 29, 30], while offering novel insights specific to T‐ALL biology. Notably, NUDT21 depletion led to downregulation of the anti‐apoptotic protein BCL‐2, suggesting that NUDT21 contributes to the anti‐apoptotic state characteristic of T‐ALL cells. Given the established role of BCL‐2 in leukemia cell survival and therapy resistance, this observation raises the possibility that T‐ALL cases with high NUDT21 expression may exhibit increased reliance on BCL‐2 signaling and could be more responsive to BCL‐2–targeted therapies.

The identification of UBE2D3 as a key downstream effector was particularly significant, as it connects NUDT21‐mediated APA regulation to the ubiquitin‐proteasome system—a pathway increasingly recognized as crucial for oncogenesis [31]. This finding suggests that NUDT21 may coordinate protein homeostasis through selective regulation of ubiquitin‐related genes. Importantly, our functional rescue experiments solidify the hierarchical relationship within this axis. The ability of UBE2D3 overexpression to rescue the proliferation and survival defects caused by NUDT21 loss establishes UBE2D3 as a principal downstream mediator. Moreover, the concomitant regulation of MYC protein levels by UBE2D3 perturbation and rescue positions MYC as a key downstream component of the NUDT21‐UBE2D3 signaling branch, essential for sustaining the oncogenic state in T‐ALL. This delineates a coherent causal pathway from NUDT21 overexpression to enhanced leukemia progression.

Our analysis reveals modest coupling between NUDT21‐mediated transcriptional regulation and APA, with 417 genes coregulated at both levels. This coordinate control likely reinforces NUDT21's oncogenic function by ensuring multi‐layered regulation of key target genes, thereby enhancing the robustness of its pro‐tumorigenic program. Beyond post‐transcriptional regulation, our data unexpectedly reveal a direct transcriptional role for NUDT21, particularly at the MYC locus. NUDT21 localizes to transcriptionally active promoters and interacts with lineage‐defining transcription factors, including ETV6, MYB, RUNX1, and GATA3, to promote MYC transcription. This expands the known functions of APA factors beyond their canonical roles in mRNA processing [7, 13, 19] and provides a potential explanation for the particularly strong oncogenic effects we observed. The physical interaction between NUDT21 and master T‐ALL transcription factors suggests a novel mechanism whereby RNA processing factors may scaffold transcriptional complexes, paralleling recent discoveries about biomolecular condensates in gene regulation [32, 33, 34, 35]. This bifunctional nature of NUDT21 – coordinating both transcriptional and post‐transcriptional regulation—represents a significant advance in understanding leukemia biology.

Our findings collectively delineate a synergistic oncogenic model in which NUDT21 drives T‐ALL progression by coordinately promoting UBE2D3 expression through APA‐mediated 3'UTR lengthening and activating MYC transcription. The therapeutic relevance of this model is underscored by the anti‐leukemic activity of ouabain octahydrate. While cardiac glycosides have previously been reported to exhibit anticancer properties [24], our study provides mechanistic evidence that ouabain induces apoptosis by triggering proteasome‐dependent degradation of NUDT21, as demonstrated by its reversal with the inhibitor MG132. Consequently, this leads to concurrent suppression of the UBE2D3/MYC axis and activation of apoptotic signaling. This discovery gains particular relevance given recent successes in targeting RNA processing machinery for cancer therapy [28, 36, 37, 38], suggesting that pharmacological modulation of APA factors may represent a viable strategy. The convergence of NUDT21's effects on both MYC transcription and UBE2D3 mRNA processing reveals an elegant, coordinated mechanism for maintaining oncogenic signaling while presenting multiple vulnerable nodes for therapeutic intervention. However, we note that ectopic overexpression of NUDT21 failed to rescue, and in J.gamma1 cells even enhanced Oua‐induced cytotoxicity, despite MG132 effectively blocking Oua‐mediated NUDT21 degradation. This suggests that while proteasomal degradation of NUDT21 is a significant event, the potent anti‐leukemic activity of Oua may involve additional, NUDT21‐independent mechanisms, consistent with the known multi‐target nature of cardiac glycosides.

Future studies should explore whether NUDT21's bifunctionality represents a broader paradigm for RNA‐binding proteins in cancer, and investigate combinatorial approaches targeting both its APA and transcriptional activities. A limitation of this study is that our mechanistic and functional investigations primarily relied on established T‐ALL cell lines due to the inherent challenges of obtaining and maintaining primary patient samples for such assays. While our findings establish the NUDT21‐UBE2D3‐MYC axis as a potent oncogenic driver in vitro and in vivo, future validation using primary T‐ALL blasts will be crucial to fully ascertain its clinical relevance and translational potential. Furthermore, while we have focused on elucidating the UBE2D3/MYC axis as a key downstream pathway, our multi‐omics data indicate that NUDT21 regulates a broader network of genes. Future studies are warranted to define the full spectrum of effectors contributing to its oncogenic function in T‐ALL.

In summary, our work defines a previously unrecognized regulatory axis integrating APA‐mediated mRNA processing with transcriptional control of MYC, thereby advancing understanding of T‐ALL biology and highlighting new opportunities for therapeutic intervention.

## 4 Methods

### 4.1 Cell Lines and Cell Culture

T‐ALL cell lines (J‐gamma1, Jurkat, Molt4, 6T‐CEM, and CCRF‐CEM) and 293FT cells were obtained from the Cell Bank of the Chinese Academy of Sciences. The T‐ALL lines were cultured in RPMI 1640 medium (Gibco) with 10% FBS (Gibco) and 1% penicillin‐streptomycin (Thermo Fisher Scientific). Meanwhile, 293FT cells were grown in DMEM (Basal Media, K211107) supplemented with 10% FBS, 2 mm L‐glutamine, and 1% non‐essential amino acids (NEAA). All cell lines used in this study underwent short tandem repeat (STR) profiling for authentication and were regularly monitored for mycoplasma contamination using a commercial detection kit (Beyotime, C0297M).

### 4.2 Cell Viability Assay

Ouabain octahydrate (MCE, HY‐B0542) was initially prepared as a 100 mm stock solution in DMSO, then diluted in culture medium to the required concentrations. For IC50 determination, cells were plated at 2 × 10^4^ cells/well and exposed to ouabain octahydrate (10‐80 nm) for 24 h. To evaluate proliferation, 2,000 cells/well were seeded and incubated with CCK‐8 reagent (APExBIO, K1018) on days 1, 3, 5, and 7. Absorbance readings at 450 nm were obtained using a Bio‐Rad microplate reader to assess viability.

### 4.3 Apoptosis Assay

Apoptosis was evaluated with the BD Annexin V‐FITC Apoptosis Detection Kit (BD Pharmingen, 556547). Puromycin‐selected cells (Sigma–Aldrich) were harvested, PBS‐washed, and resuspended in binding buffer. Cell suspensions were stained with Annexin V‐FITC and PI (5 µL each, 15 min, RT, dark), then diluted with 300 µL binding buffer. Flow cytometry was performed within 1 h using a Beckman Gallios system, with FlowJo analysis quantifying Annexin V+/PI‐ (early apoptotic) and Annexin V+/PI+ (late apoptotic/necrotic) populations.

### 4.4 Cell Cycle Analysis

For cell cycle analysis, stable transfectants selected with puromycin (Sigma–Aldrich) were initially pelleted by low‐temperature centrifugation (1,000 rpm, 5 min, 4°C). Cellular fixation was achieved through ethanol treatment (75%, −20°C, overnight). Samples were then subjected to propidium iodide staining (0.05 mg/mL in 0.1% sodium citrate/Triton X‐100 solution) with 37°C incubation for 30 min (light‐protected). Flow cytometric evaluation was performed using a Beckman Gallios system, with subsequent cell cycle distribution analysis conducted via FlowJo software.

### 4.5 Soft Agar Colony Formation Assay

A biphasic agar system was prepared using 0.7% and 1.2% agarose (Sigma, A9045‐10G) in 2× RPMI 1640 (Yuchun, YC‐1010) supplemented with 20% FBS and antibiotics (penicillin 100 U/mL, streptomycin 100 µg/mL). The 1.2% agarose‐medium mixture was first allowed to solidify in 6‐well plates as the base layer. Cells (1 × 10^3^) suspended in 0.7% agarose‐medium mixture were then plated as the top layer. Cultures were incubated at 37°C/5% CO_2_ with triweekly medium changes. After 2–3 weeks, colonies were paraformaldehyde‐fixed (4%, Beyotime, P0099), stained with Giemsa (0.1%, Beyotime, C0131), and enumerated microscopically (≥50 µm diameter).

### 4.6 Western Blot

Protein lysates (RIPA buffer, Beyotime P0013) were quantified and separated on precast gels (GenScript M42015C). After PVDF transfer (Merck Millipore IPVH00010), membranes were blocked (5% milk/TBST, 1 h), incubated with primary (overnight, 4°C) and HRP‐secondary (1 h, RT) antibodies, then developed with ECL. Antibody information appears in Table S2.

### 4.7 Lentivirus Production and Infection

Short hairpin RNAs (shRNAs) targeting NUDT21, UBE2D3, GATA3, MYB, and RUNX1 were cloned into the pLKO.1‐puro vector (IGE Biotechnology). Lentiviral particles were produced in HEK293FT cells by co‐transfection with the packaging plasmids psPAX2 and pMD2.G (Addgene), concentrated by ultracentrifugation, and titrated by quantitative PCR. T‐ALL cells were infected with lentivirus in the presence of polybrene (1 µg/mL; Sigma‐Aldrich) for 24 h and subsequently selected with puromycin (2 µg/mL) for 72 h.

For ectopic expression of NUDT21, a Flag‐tagged NUDT21 coding sequence was cloned into the pLKO.1‐puro vector (Tongyong Biotechnology). For UBE2D3 overexpression, an HA‐tagged UBE2D3 coding sequence was cloned into the pLKO.1‐G418 vector (IGE Biotechnology). Lentiviral particles for overexpression constructs were generated in HEK293FT cells using PEI‐mediated transfection with a total of 15 µg plasmid DNA at a target: psPAX2: pMD2.G ratio of 2:2:1. T‐ALL cells were transduced with the resulting lentiviruses and selected with puromycin or G418, respectively, to establish stable overexpression cell lines. All shRNA and ectopic expression target sequences are provided in Tables S3 and S5.

### 4.8 RNA Isolation and Quantitative Real‐Time PCR (qRT‐PCR)

RNA was extracted (FastPure Kit, Vazyme RC112‐01) and quantified (NanoDrop, Thermo Fisher). High‐quality samples (A260/A280 = 1.8–2.0) were reverse transcribed (1 µg RNA; HiScript III, Vazyme R323‐01). qPCR was performed in triplicate (SYBR Green Master Mix, Roche 04707516001; LightCycler 480 II). Gene expression (2‐ΔΔCt method) was normalized to GAPDH. See Table S4 for primers.

### 4.9 Dual Luciferase Reporter Assay

The vector UBE2D3‐short or UBE2D3‐long with mutated proximal poly(A) signal sequences was generated (Table S5). Then plasmids were transfected into T‐ALL cells (J.gamma1, Jurkat) using the ProteanFect Max Transfection Reagent Kit (PT01‐0030A, Xihu Juhe, China). After transfection for 48 h, relative luciferase activity was detected with a Dual‐Luciferase Reporter Assay kit (DL101‐01, Vazyme Biotech) according to the user's manual. Firefly luciferase activity was detected and used to normalize Renilla luciferase activity.

### 4.10 Animal Experiments

All animal experiments were conducted following approval by Soochow University's IACUC (Protocol CAM‐SU‐AP: JP‐2018‐1) in accordance with institutional animal welfare guidelines. Female NSG mice (NOD.Cg‐*Prkdc^scid^Il2rg^em1Smoc^*; 6‐week‐old; n = 5/group) were administered luciferase‐tagged J.gamma1 cells (4 × 10^6^ cells in PBS) via tail vein injection. For knockdown experiments, cells were pre‐transduced with NUDT21‐targeting or scrambled shRNA. Tumor development was assessed weekly through bioluminescent imaging (IVIS Spectrum, PerkinElmer) post‐D‐luciferin administration (150 mg/kg, i.p.). Humane endpoints were implemented upon manifestation of distress symptoms, with CO2 asphyxiation employed for euthanasia. Collected tissues were subjected to histopathological (H&E), immunohistochemical, and flow cytometric analyses using CD45 antibody (BioLegend, 304008, 1:100). Daily health evaluations were performed by veterinary personnel, with predetermined experimental endpoints to minimize discomfort.

### 4.11 RNA Sequencing and Analysis

RNA isolation was performed according to the manufacturer's instructions (Novozymes, RC112‐01). Sequencing services were provided by Ruixing Bio‐Information Technology Co., Ltd. (Wuhan, China). Raw sequencing reads were processed using fastp (v0.23.1), followed by alignment to the hg38 reference genome through STAR (version 2.7.10a) with Gencode annotations. Differential expression analysis was carried out employing DESeq2 (version 1.40.2), with significantly differentially expressed genes (DEGs) defined as those meeting the criteria of adjusted p‐value <0.01 and fold change >1.2 or <0.83.

### 4.12 PAS Sequencing and Analysis

Ruixing Bio—Information Technology Co., Ltd. (Wuhan, China) conducted PAS‐seq library construction and high‐throughput sequencing. Total RNA was extracted using the FastPure Cell/Tissue Total RNA Isolation Kit V2 (Vazyme, RC112‐01). For each sample, 5 µg of total RNA was treated with RQ1 DNase (M6101, Promega, USA) to remove DNA, and fragmented RNAs were used for PAS‐seq library preparation. mRNAs were captured with the mRNA Capture Beads kit (N401, Vazyme, China). Directional PAS‐seq libraries were constructed from fragmented mRNA samples employing the KAPA Stranded mRNA‐Seq Kit (Roche, KK8544) compatible with Illumina sequencing systems. Subsequently, the prepared libraries were analyzed through paired‐end sequencing. PAS‐seq libraries were constructed from two independent biological replicates for both control and NUDT21‐knockdown J.gamma1 cells. Both of the two biological replicates were used to calculate the differential usage of proximal and distal poly(A) sites between the two conditions.

The detailed analysis pipeline is as follows. Only read 2 was used to determine the polyA sites. Raw reads were trimmed to remove adapters, low‐quality bases with a score below 20, more than 3 consecutive “G”s at the end of the sequence, as well as “N”s and all subsequent sequences. Reads containing no less than 30% of the bases with a quality score below 20 were filtered out. Processed reads with a remaining length greater than 20 were retained. To remove poly(A) tract, we first search for at least 8 consecutive A's from the end of the reads. If more than 2 consecutive non‐A bases are encountered, the poly(A) tract and all subsequent bases are removed, retaining only those reads with a length of 12 bases or more. Next, we scan for A's starting from the 5' end of the reads; if more than 9 consecutive A's are present, the poly(A) tract and all subsequent bases are removed, with reads of 12 bases or longer retained. The processed reads were then aligned to hg38 genome using HISAT2 (version 2.2.1) with Gencode gene annotation. Effectively aligned reads’ 3’ ends were defined as the candidate pA sites. The candidate sites were then clustered within 24 nt, and the sites with the most reads supporting were taken as the pA sites. For the subsequent APA analysis, we only used the pA sites with polyadenylation signal (AAUAAA, AUUAAA, AGUAAA, UAUAAA, AAUACA, CAUAAA, AAUAUA, GAUAAA, AAUGAA, AAGAAA, ACUAAA, AAUAGA, AAUAAU, AACAAA, AUUACA, AUUAUA, AACAAG, AAUAAG) within 10–40 nt upstream and located in annotated genes. The Relative Expression Difference (RED) was computed as the log2 ratio difference in isoform abundance (dPAS vs. pPAS) between comparative samples. APA events were considered significant at |RED| > log2(1.5) with B‐H adjusted p‐values <0.05 (Fisher's exact test). Gene functional enrichment was analyzed with the clusterProfiler package (version 4.2.2).

### 4.13 APA Analysis of Clinical Samples

We utilized the DaPars (https://github.com/ZhengXia/DaPars) algorithm to identify alternative polyadenylation and calculate the percentage of distal polyA site usage index (PDUI) for each gene. Significant APA events were identified with the adjusted *p* value < 0.05.

### 4.14 eCLIP‐seq Experiment, Library Preparation and Sequencing

eCLIP‐seq experiment and high through‐put sequencing and data analysis were conducted by Seqhealth Technology Co., LTD., Wuhan, China. J.gamma1 cells were processed following comprehensive procedures adapted from established protocols [39, 40]. Due to experimental constraints, one replicate was generated for initial binding site identification. Raw reads were processed by fastp (version 0.23.1) in first, and the processed reads were then corrected and deduplicated based on the UMI. After rRNA removal, the clean reads were mapped to the hg38 reference genome. MACS2 was used to call peaks with the following parameters: ‐f BAM ‐B –bw 50 –mfold 2 50 –slocal = 1000 –llocal = 10000 –keep‐dup = 1 –qvalue 0.05. Then Homer was used to find motif enrichment.

### 4.15 Cleavage Under Targets and Tagmentation (Cut &Tag) Assay

The Cut &Tag experiment was conducted in two independent biological replicates in J.gamma1 cells utilizing the Hyperactive Universal Cut &Tag Assay Kit (Vazyme, TD903‐01) following the guidelines provided by the manufacturer. 2 × 10^6^ cells were used for this assay. Detailed experimental procedures refer to the literature [23], and the primary antibody used was NUDT21 (Proteintech,10322‐1‐AP). Raw reads were firstly processed by fastp (version 0.23.1), and then clean reads were aligned against the hg38 reference genome using the Bowtie2 (version 2.2.5) algorithm. Duplicate reads were then removed using Picard tools, and peaks were called via MACS3. Gene functional enrichment analysis was performed using Enrichr (https://maayanlab.cloud/Enrichr/).

### 4.16 ChIP‐qPCR Analysis

To determine whether NUDT21 is required for the occupancy of lineage‐specific transcription factors (TFs) at target gene promoters, we performed chromatin immunoprecipitation followed by quantitative PCR (ChIP‐qPCR). Briefly, chromatin was cross‐linked and isolated from control (shNC) and NUDT21‐knockdown (shNUDT21) T‐ALL cells. Immunoprecipitation was carried out using antibodies specific for ETV6, MYB, RUNX1, and GATA3, with IgG serving as a negative control. Antibody information appears in Table S2. The precipitated DNA was analyzed by qPCR using primers targeting the *MYC* promoter region. See Table S4 for *MYC* promoter primers. Enrichment was calculated relative to the IgG control.

### 4.17 Statistical Analysis

Statistical comparisons were conducted through unpaired two‐tailed Student's *t*‐tests or one‐way ANOVA where applicable, while survival analyses employed Mantel‐Cox log‐rank methodology. All computations were performed using GraphPad Prism software (version 9.2.0). Results reflect three independent biological replicates demonstrating reproducible outcomes. Statistical significance was defined as follows: ^*^ *p* < 0.05, ^**^ *p* < 0.01, ^***^ *p* < 0.001, ^****^ *p* < 0.0001.

### 4.18 Ethics Statement

Ethical approval for clinical research was obtained from the Institutional Review Board at Children's Hospital of Soochow University (CHSU‐IRB‐2023‐45). Animal experimentation protocols were reviewed and approved by the Institutional Animal Care and Use Committee of Soochow University (CAM‐SU‐AP#: JP2018‐1).

## Author Contributions

The research was conducted by C.L.Q., Q.J., Y.J.W., C.X.S., and Z.Z., while data analysis was carried out by C.L.Q. and Y.Y. The study was conceptualized by J.W.W., Y.Y., S.Y.W., and J.P. Methodology development was undertaken by M.Z., J.W.W., Y.Y., and J.P., and the investigation was performed by C.L.Q., Z.H.L., H.Z., Q.Y.Z., Q.J., S.L.Z., and J.J.Y. The original draft of the manuscript was prepared by Y.Y. and S.Y.W., with subsequent review and editing conducted by Y.Y., S.Y.W., and J.P. Supervision was provided by Y.Z.L., Z.J.B., Y.F.T., S.Y.H., and J.P. Funding for the project was acquired by Y.Z.L., S.Y.H., J.P., J.W.W., and S.Y.W.

## Conflicts of Interest

The authors declare no conflicts of interest.

## Supporting information




**Supporting File 1**: advs75118‐sup‐0001‐FigureS1‐12.docx.

**Supporting File 2**: advs75118‐sup‐0002‐TableS1.docx.

**Supporting File 3**: advs75118‐sup‐0003‐TableS2‐16.xlsx.

## Data Availability

The data that support the findings of this study are available on reasonable request from the corresponding author. PAS‐seq (accession number GSE305440), eCLIP‐seq (accession number GSE305851), RNA‑seq (accession number GSE305438), and CUT‐Tag (accession number GSE305852) data have been submitted to the GEO database. Public datasets are available from the GEO database, including GSE59657 (Jurkat ChIP‐seq), GSE76783 (CCRF‐CEM ChIP‐seq), GSE267758 (J.gamma1 ChIP‐seq), GSE59657 (MOLT3 ChIP‐seq), GSE79288 (MOLT4 ChIP‐seq), GSE243772 (PF382 ChIP‐seq), GSE231486 (CD34+ HSC ChIP‐seq), and GSE267451 (RUNX1 CUT&Tag).
