# Supplementary material for: NUDT21 Drives T‐Cell Acute Lymphoblastic Leukemia Through Dual Regulation of Alternative Polyadenylation and Transcriptional Activation
Source: Adv Sci (Weinh). 2026 Apr 2;13(34):e20693. doi: 10.1002/advs.202520693 (PMC13285137; doi:10.1002/advs.202520693)
Supplement: Supplementary file 1 — Supporting File 1: advs75118‐sup‐0001‐FigureS1‐12.docx. [file ADVS-13-e20693-s003.docx]

**Supporting Information**


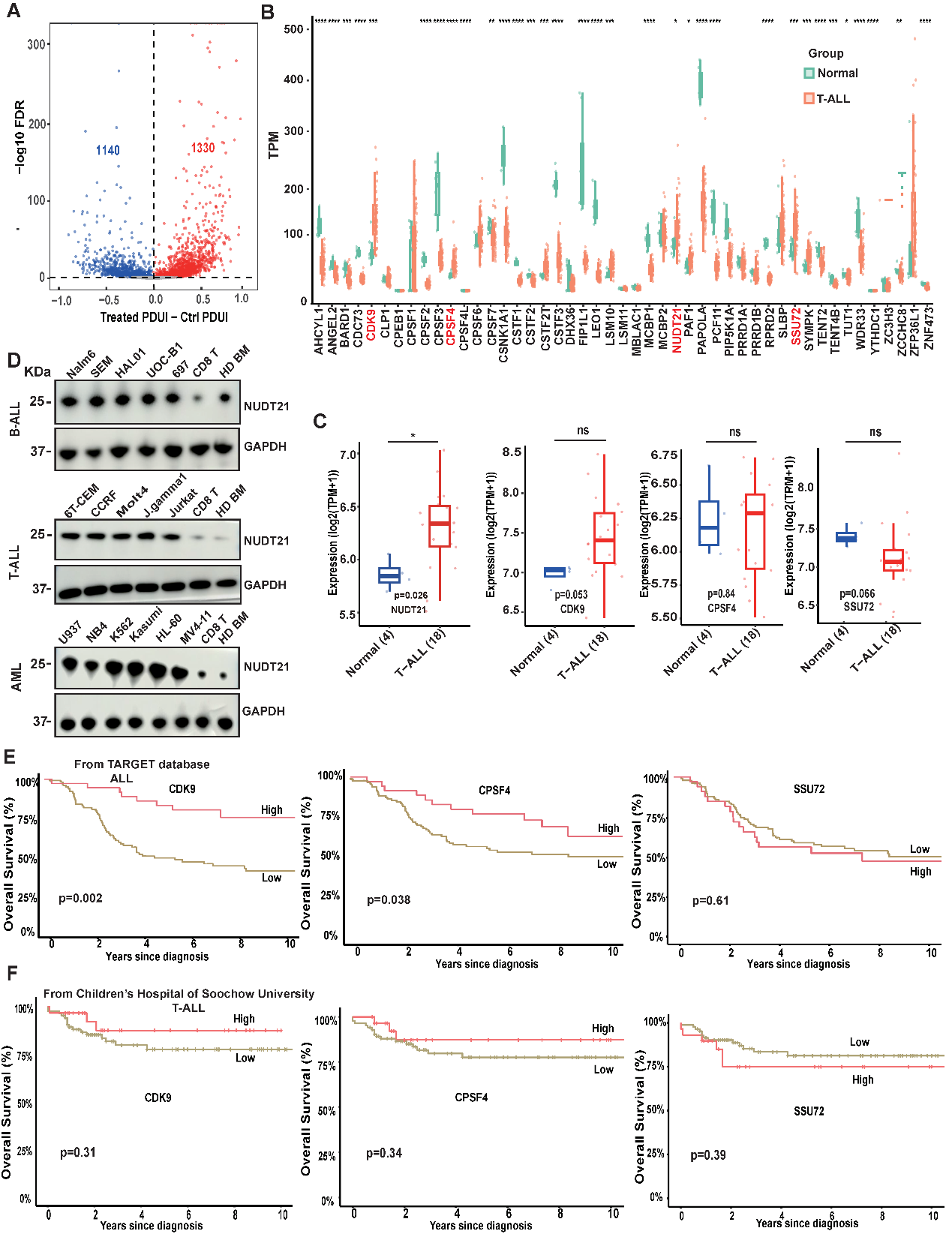


**Figure S1. Screening and validation of polyadenylation factors in T-ALL biology. A.** Volcano plot displaying statistically significant 3'UTR-shortened and -lengthened genes (FDR < 0.05). **B.** Boxplot of mRNA expression levels of polyadenylation-related genes across T-ALL patients-derived cells (n=60) and normal thymus cells (n=8) based on the datasets of GSE110637 and SRP325613. **C.** Expression of NUDT21, CDK9, CPSF4 and SSU72 in normal and T-ALL patients-derived cells from the dataset of GSE146901. P value is indicated in the figures. **D.** Expression of NUDT21 protein was detected in the leukemia cell lines and normal sample (CD8 T cells and bone marrow of health donor (HD BM)). **E.** Correlation between CDK9, CPSF4 and SSU72 expression and overall survival was determined in TARGET database by Kaplan–Meier analysis. **F.** Correlation between CDK9, CPSF4 and SSU72 expression and overall survival was determined in our cohorts by Kaplan–Meier analysis. Error bars represent three independent experiments, * p < 0.05, **p < 0.01, *** p < 0.001.


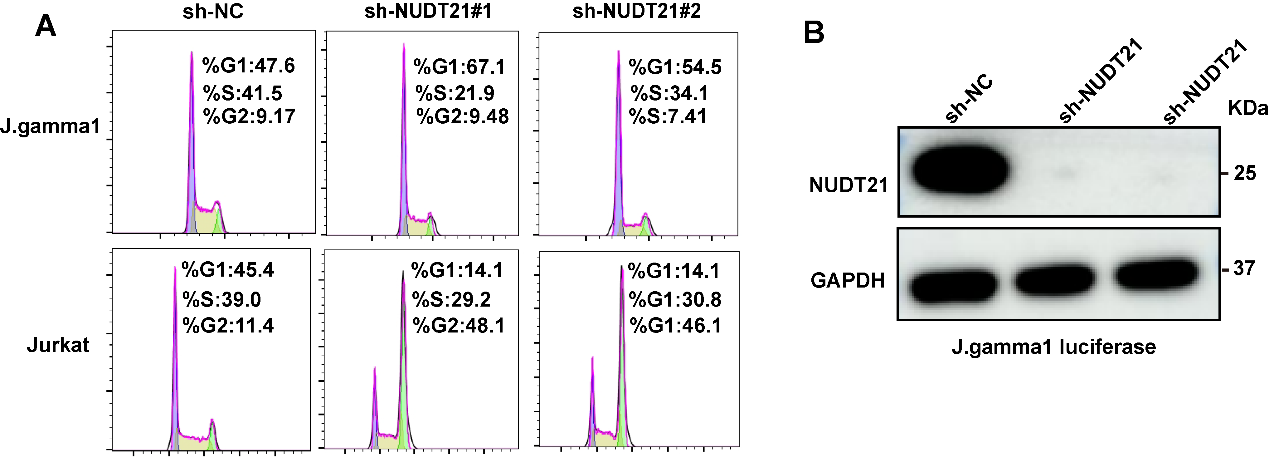


**Figure S2. NUDT21 knockdown validation and cell cycle perturbations. A.** Presentation of cell cycle distribution. **B.** the validation of NUDT21 knockdown in the cells transfected with Luciferase vector by Western blot.


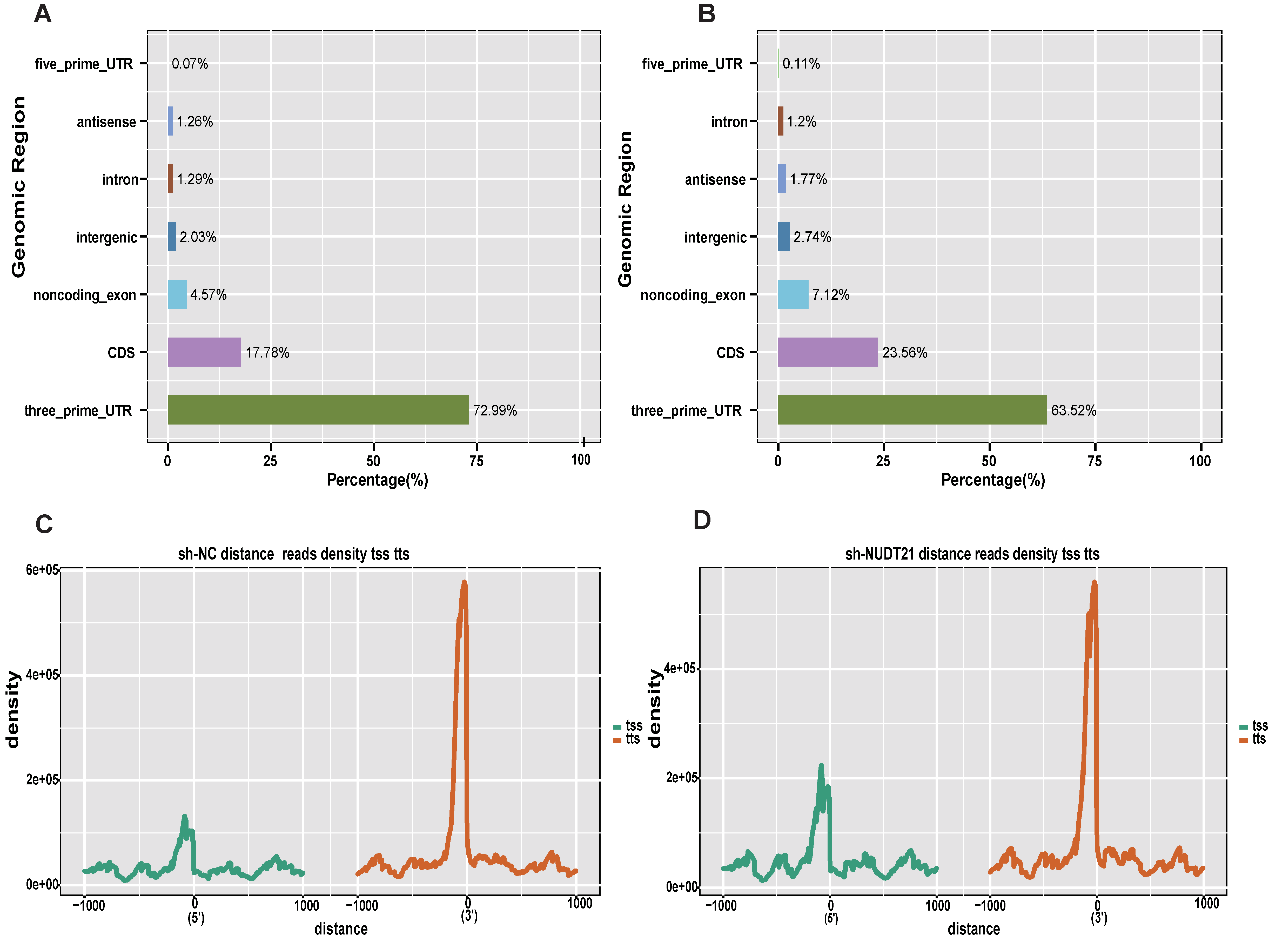


**Figure S3. PAS-seq data quality metrics. A.** Mapping distribution of reads across genomic regions in sh-NC group**.** **B.** Mapping distribution of of reads across genomic regions in sh-NUDT21 group**. C.** Distribution of reads around transcription start sites (TSS) and transcription termination sites (TTS) in sh-NC group. **D.** Distribution of reads around transcription start sites (TSS) and transcription termination sites (TTS) in sh-NUDT21 group.


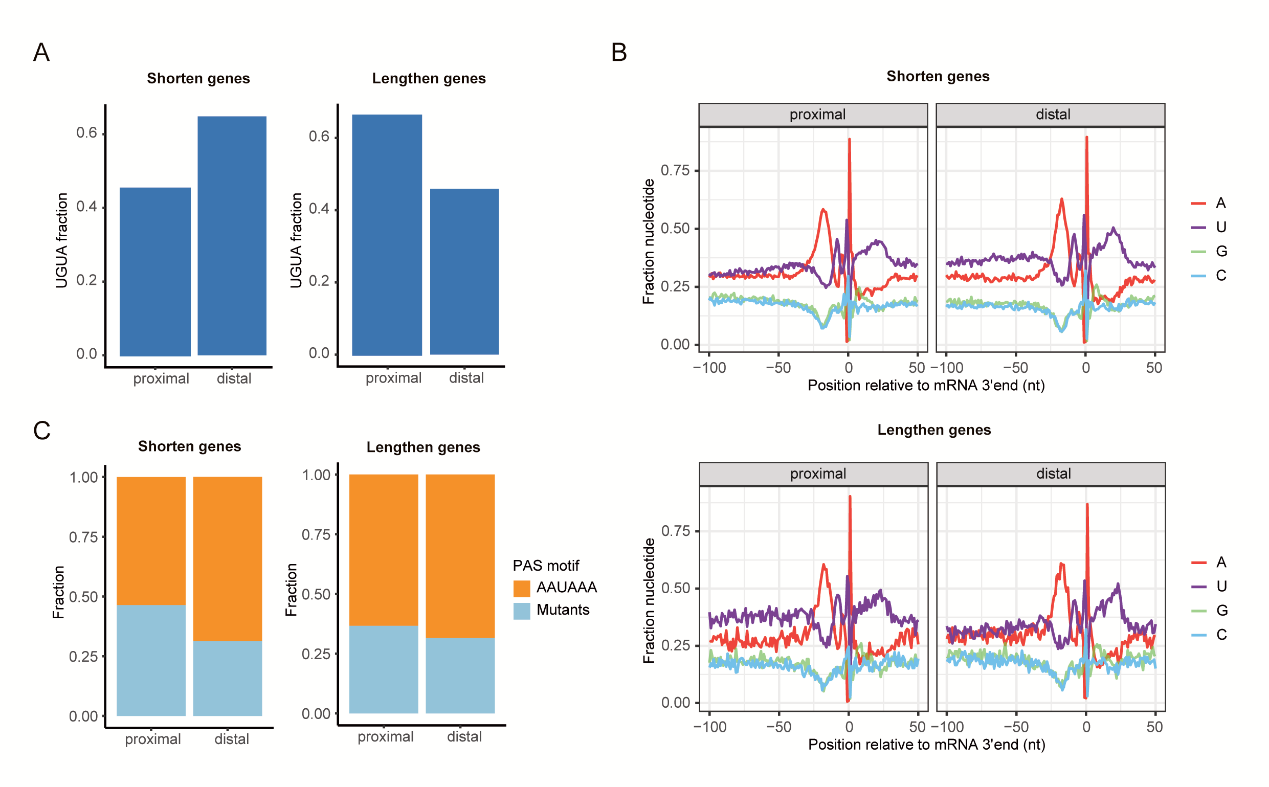


**Figure S4. Sequence features of APA genes regulated by NUDT21. A.** Fraction of UGUA motif within 100 nt upstream of the proximal and distal polyA sites for APA genes shortening and lengthening after NUDT21 knockdown. **B.** Nucleotide distribution upstream 100 nt and downstream 50 nt of the proximal and distal polyA sites for APA genes shortening and lengthening after NUDT21 knockdown. **C.** Fraction of AAUAAA or its 1-nt variants within 10 ~ 40 nt upstream of the proximal and distal polyA sites for APA genes shortening and lengthening after NUDT21 knockdown.


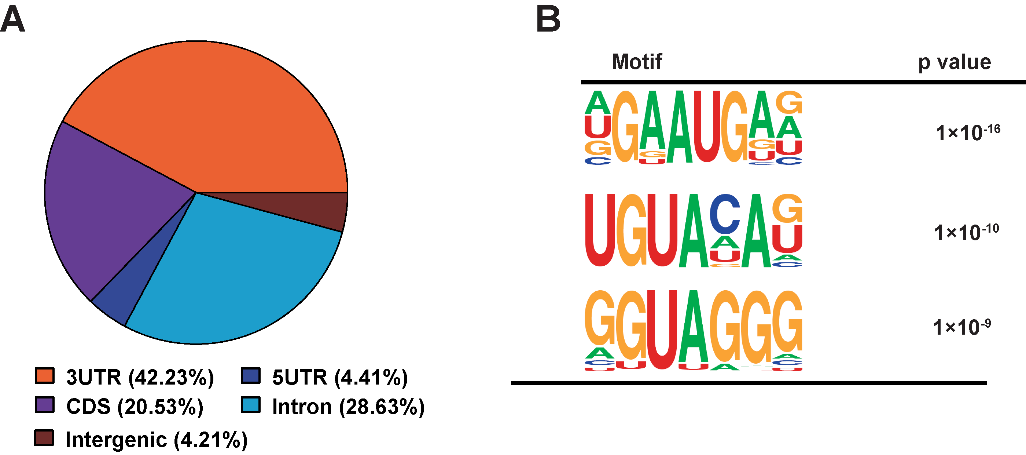


**Figure S5. eCLIP-seq peak calling and motif discovery. A.** Pie-chart showing genomic distribution of eCLIP-seq peaks in J.gamma1 cells. **B.** NUDT21 binding motifs identified based on eCLIP-seq peaks.


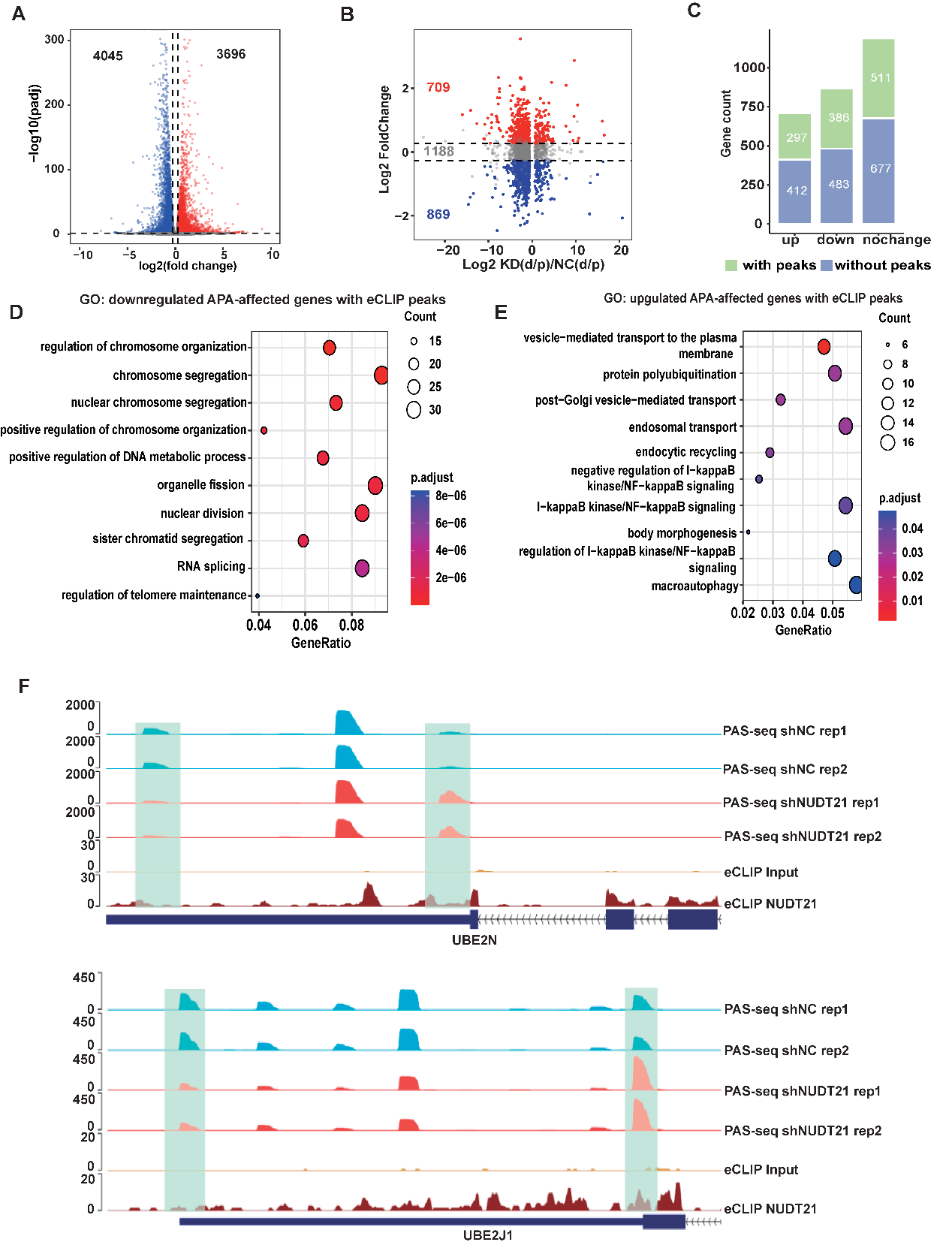


**Figure S6. Integrated analysis of gene expression, APA, and NUDT21 binding. A.** Volcano map showing the differentially-expressed genes (DEGs) by RNA-seq following NUDT21 knockdown in J.gamma1 cells (Fold Change >1.2 or < 0.83, FDR<0.01). The counts of upregulated and downregulated genes are indicated in the figure. **B.** Correlation between relative expression difference (RED) value and fold change in gene expression after NUDT21 knockdown is shown. The counts of APA-regulated genes in upregulation, downregulation and non-changing are indicated in the figure. **C.** Barplot showing the counts of up-regulated, down-regulated, and non-changing genes with NUDT21 binding sites in their 3'UTRs. Gene counts for each group are indicated in the figure. **D.** Top 10 enriched GO terms for downregulated genes with eCLIP-seq peaks located in the 3’UTR but without APA events. **E.** Top 10 enriched GO terms for upregulated genes with eCLIP-seq peaks located in the 3’UTR but without APA events. **F.** Genome browser plots showing the PAS-seq and eCLIP-seq signals around UBE2N and UBE2J1 3’UTR locus.


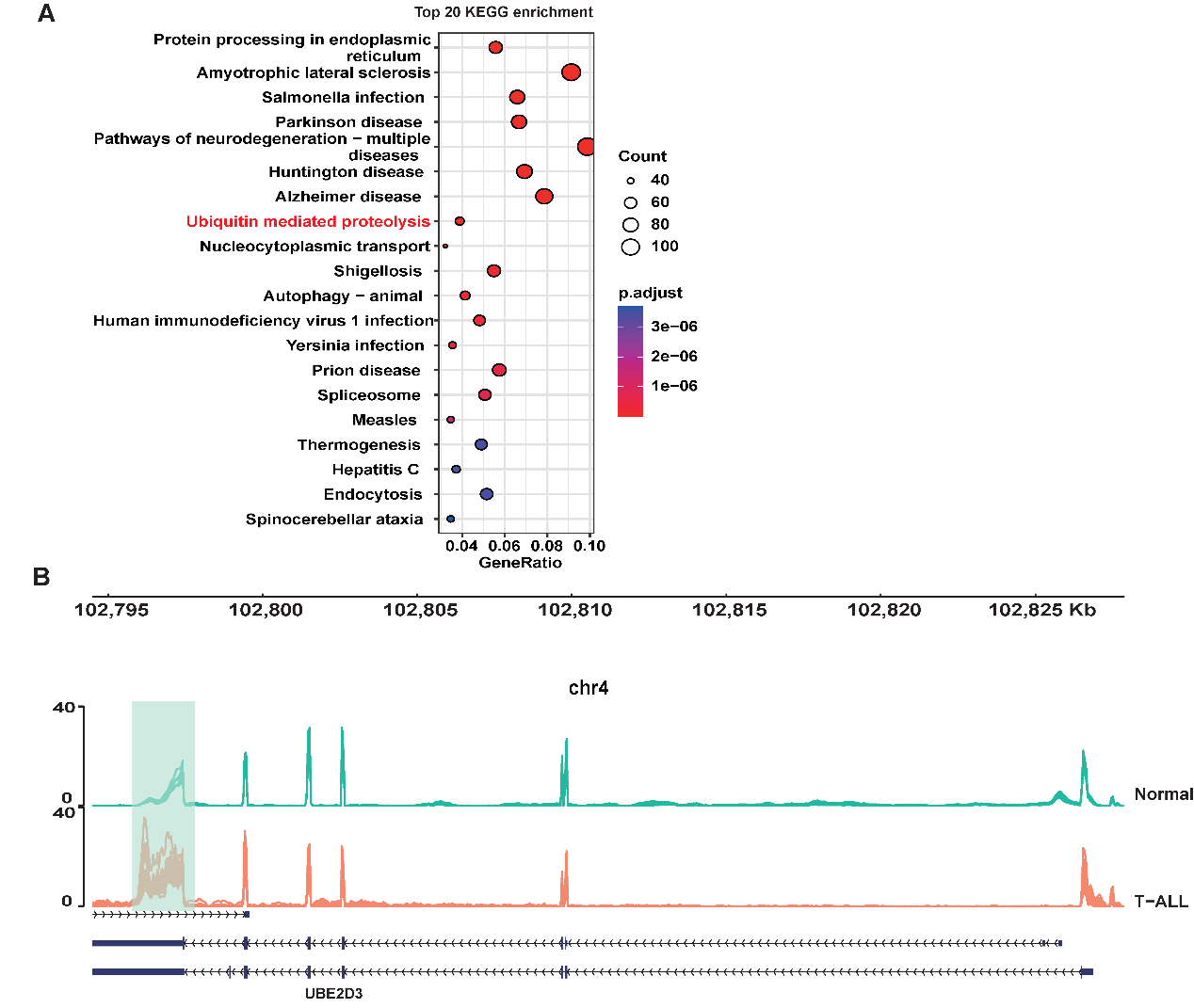


**Figure S7. Functional and mechanistic insights into APA dysregulation in T-ALL. A.** Top 20 enriched KEGG pathways of genes with significant APA events in T-ALL compared to normal samples. **B.** RNA-seq signals near UBE2D3 in T-ALL and normal samples. The region of 3’UTR is highlighted.


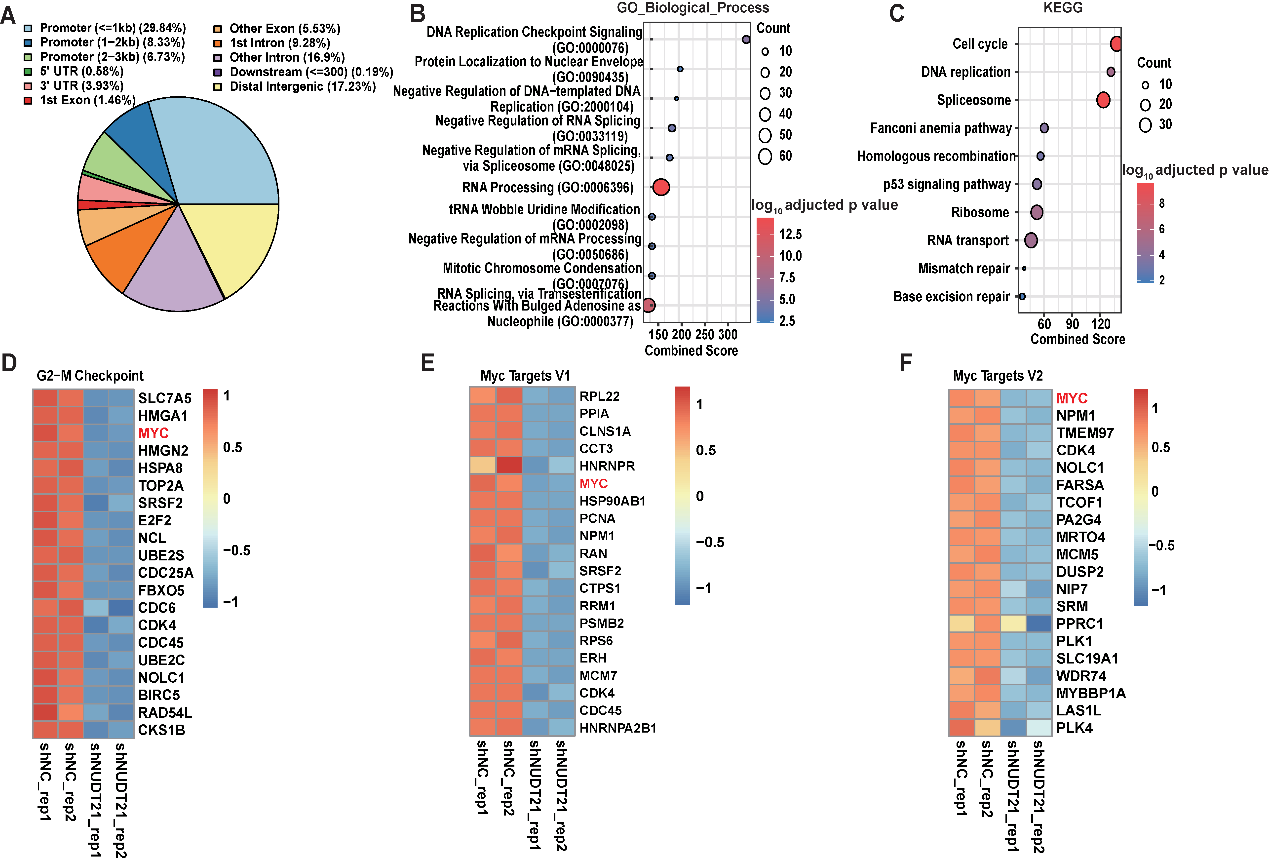


**Figure S8. NUDT21 chromatin occupancy and functional enrichment following NUDT21 depletion. A.** Peak distribution in the genome for NUDT21 chromatin occupancy profile by Cut &Tag. **B and C.** Bubble plot showing the top 10 enriched GO biological processes and KEGG pathways of DEGs following NUDT21 knockdown in J.gamma1 cells. **D.** Heatmap showing the top 20 downregulated genes in the dataset of G2-M checkpoint. **E.** Heatmap showing the top 20 downregulated genes in the dataset of MYC targets v1. **F.** Heatmap showing the top 20 downregulated genes in the dataset of MYC targets v2.


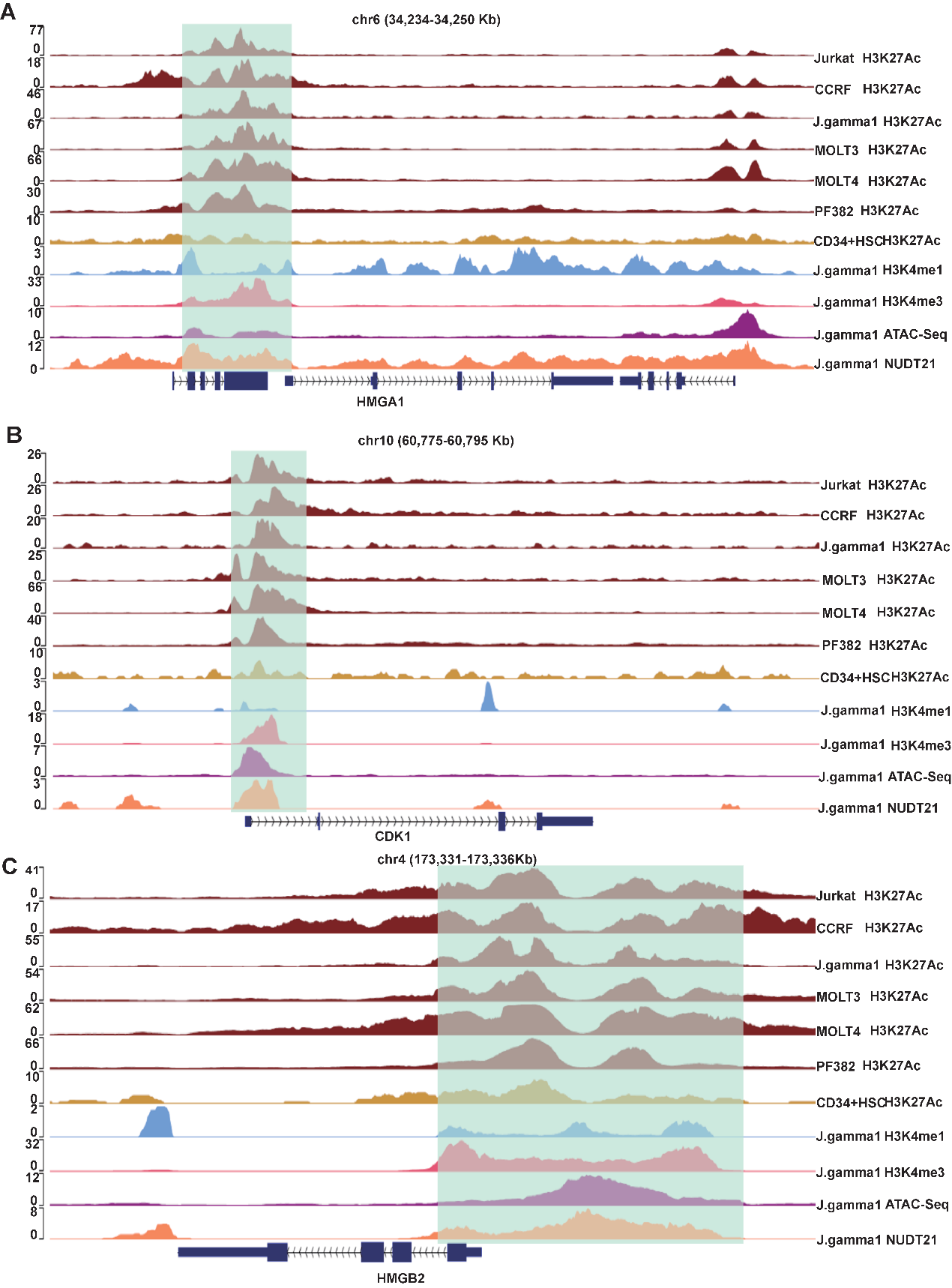
**Figure S9. NUDT21 occupies active promoter hubs marked by H3K27ac/H3K4me3 and chromatin accessibility. A.** Signals of H3K27ac/H3K4me3/H3K4me1 ChIP-seq across cell lines, ATAC-seq in J.gamma1, and NUDT21 Cut &Tag in J.gamma1 at the HMGA1 promoter. **B.** Signals of H3K27ac/H3K4me3/H3K4me1 ChIP-seq across cell lines, ATAC-seq in J.gamma1, and NUDT21 Cut &Tag in J.gamma1 at the CDK1 promoter. **C.** Signals of H3K27ac/H3K4me3/H3K4me1 ChIP-seq across cell lines, ATAC-seq in J.gamma1, and NUDT21 Cut &Tag in J.gamma1 at the HMGB2 promoter**.**


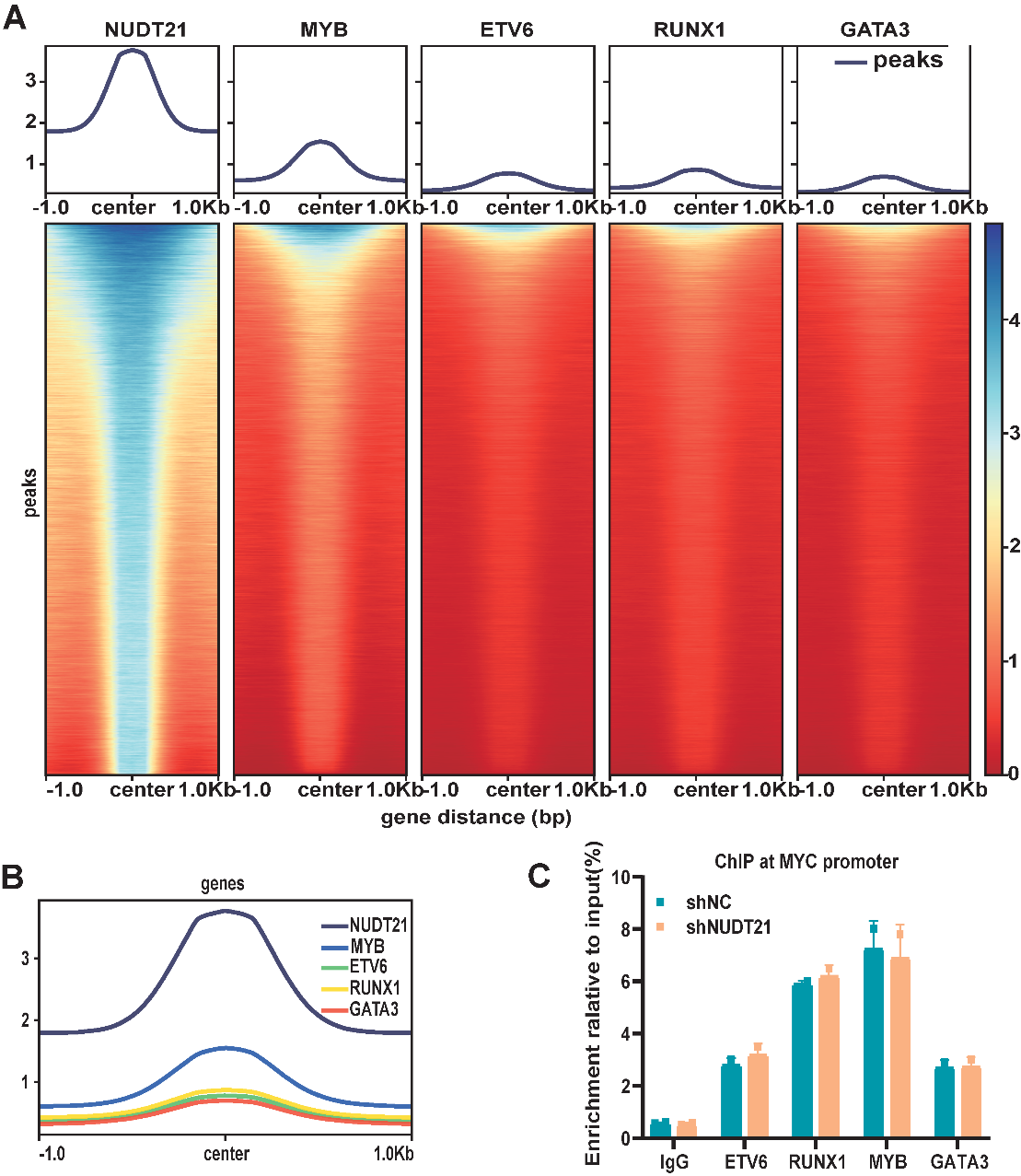


**Figure S10. Nuclear localization of NUDT21 and TFs. A and B.** The signal intensity distribution of MYB, ETV6, GATA3, and RUNX1 around the center of NUDT21 peaks. **C.** ChIP-qPCR analysis showing the enrichment of the indicated transcription factors (ETV6, MYB, RUNX1, GATA3) at the *MYC* promoter region in control (shNC) and NUDT21-knockdown (shNUDT21) T-ALL cells. Data are presented as mean ± SEM.


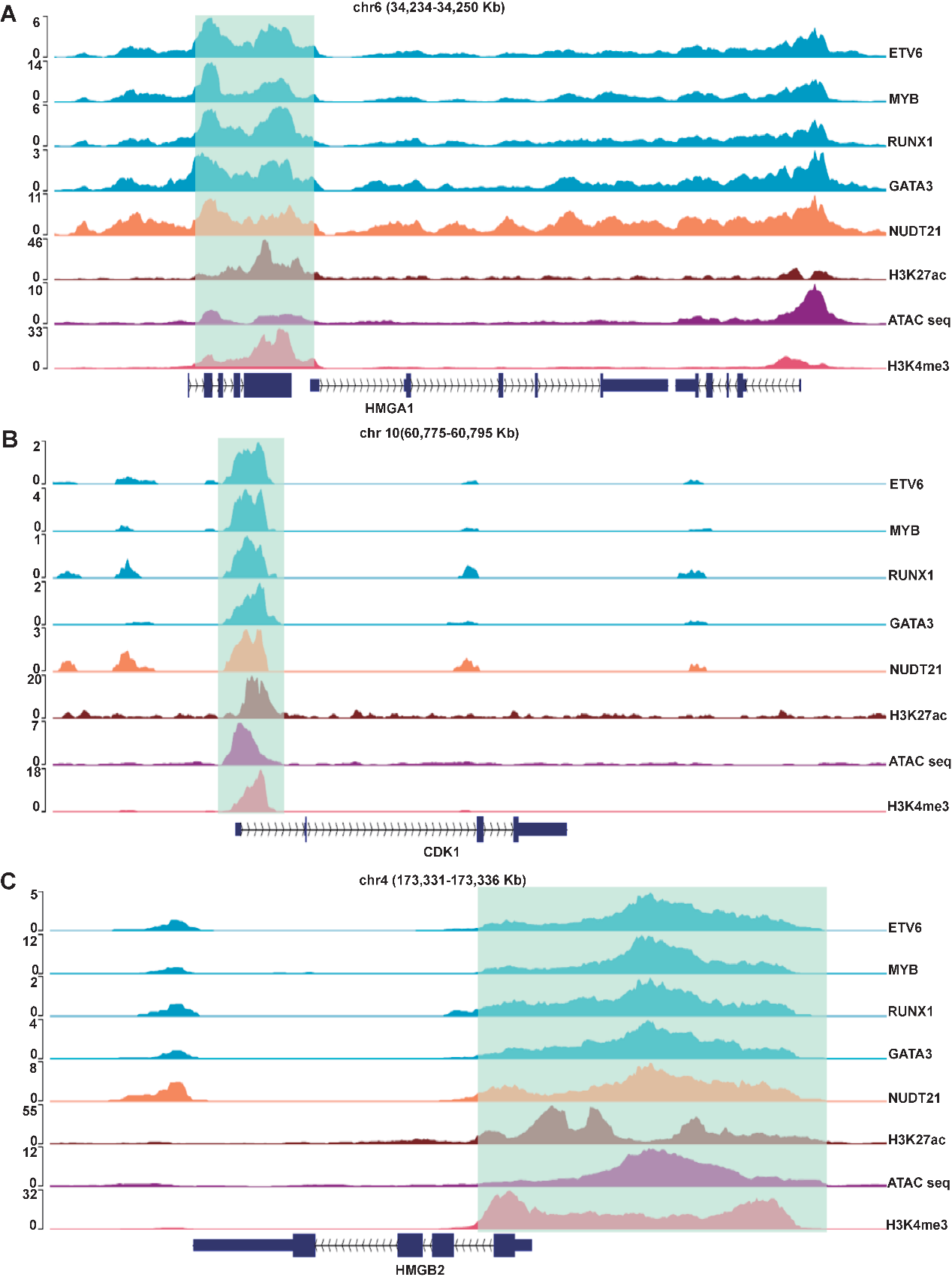


**Figure S11.   NUDT21 and master T-ALL regulators binding at target promoters. A.** Genome browser showing the Cut &Tag signals of NUDT21, ETV6, RUNX1, GATA3, and MYB at the HMGA1 region. **B.** Genome browser showing the Cut &Tag signals of NUDT21, ETV6, RUNX1, GATA3, and MYB at the CDK1 promoter region. **C.** Genome browser showing the Cut &Tag signals of NUDT21, ETV6, RUNX1, GATA3, and MYB at the HMGB2 promoter region. The region near the promoter is highlighted in the figures.


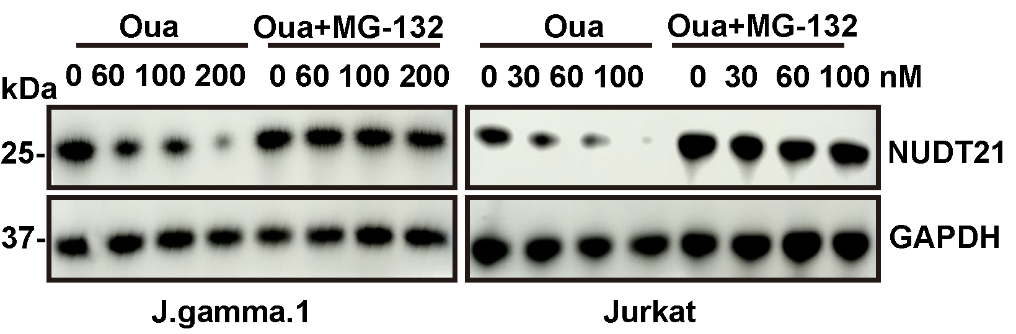


**Figure S12. MG-132 reverses ouabain-induced NUDT21 degradation in T-ALL cell lines.** 20 μM MG-132 (HY-13259) was applied to J.gamma1 and Jurkat cells, which had been treated with different concentrations of Ouabain for 12 hours, for a duration of 6 hours. The protein levels of NUDT21 and GAPDH were measured by western blotting.
