# Supplementary material for: NUDT21 Drives T‐Cell Acute Lymphoblastic Leukemia Through Dual Regulation of Alternative Polyadenylation and Transcriptional Activation
Source: Adv Sci (Weinh). 2026 Apr 2;13(34):e20693. doi: 10.1002/advs.202520693 (PMC13285137; doi:10.1002/advs.202520693)
Supplement: Supplementary file 2 — Supporting File 2: advs75118‐sup‐0002‐TableS1.docx. [file ADVS-13-e20693-s002.docx]

**Supporting Information**

**Title: NUDT21 Drives T-Cell Acute Lymphoblastic Leukemia through Dual Regulation of Alternative Polyadenylation and Transcriptional Activation**

Conglian Qiu, Jianwei Wang, Zhiheng Li, Qi Ji, Hui Zhang, Qinyi Zhang, Senlin Zhang, Juanjuan Yu, Yanfang Tao, Yijun Wu, Chunxia Shi, Zong Zai, Zimu Zhang, Yizhen Li, Zhenjiang Bai, Shaoyan Hu, Jian Pan, Yang Yang, Shuiyan Wu

Corresponding author:

Shuiyan Wu, Pediatric Intensive Care Unit, Children’s Hospital of Soochow University, Suzhou, Jiangsu, China. Tel: 86-0512-80692300; E-mail: wushuiyany@163.com

Panjian, Institute of Pediatric Research, Children’s Hospital of Soochow University, Suzhou, Jiangsu, China. Tel: 86-15150137126; E-mail: panjian2008@163.com; panjian2019@suda.edu.cn

Yang Yang, Institute of Pediatric Research, Children’s Hospital of Soochow University, Suzhou, Jiangsu, China. Tel: 86-0512-80691500; E-mail: [yy20120814@163.com](mailto:yy20120814@163.com)

**Table S1.** Analysis of univariable and multivariable cox regression for 5-years OS in pediatric T-ALL patients.

|  | **5-year OS** | | | |
| --- | --- | --- | --- | --- |
|  | **Univariable analysis** | | **Multivariable analysis** | |
| **Factors** | **HR (95% CI)** | **P value** | **HR (95% CI)** | **P value** |
| Male vs female | 1.432（0.417-4.919） | 0.569 |  |  |
| Age at diagnosis (≥10 years vs＜10 year) | 0.761（0.273-2.113） | 0.600 |  |  |
| WBC≥100×109/L | 2.002（0.760-5.271） | 0.160 | 1.828(0.690-4.845) | 0.225 |
| Hemoglobin≤100g/L | 0.547（0.215-1.391） | 0.205 |  |  |
| Platelet≤50×109 | 1.559（0.627-3.877） | 0.339 |  |  |
| BM blasts≥90% | 1.422（0.559-3.618） | 0.460 |  |  |
| ETP vs non-ETP | 1.564 (0.227–13.690) | 0.511 |  |  |
| SIL-TAL1 yes/no | 0.976(0.351-2.715) | 0.963 |  |  |
| HOX11L2 yes/no | 0.675(0.156-2.926) | 0.600 |  |  |
| MLL-r yes/no | 0.546(0.073-4.092) | 0.556 |  |  |
| CNSL yes/ no | 0.320(0.043-2.408) | 0.269 |  |  |
| NOTCH1/FBXW7 status | 0.574（0.226-1.549） | 0.244 |  |  |
| Steroid response poor vs good | 1.593(0.455-5.571) | 0.466 |  |  |
|  |  |  |  |  |
| BM not CR after induction | 0.846(0.344-2.083) | 0.717 |  |  |
| NUDT21 | 2.537（1.018-6.325） | 0.046 | 2.364(0.942-5.932) | 0.067 |

Abbreviations: HR, hazard ratio; WBC, white blood cell; BM, bone marrow; ETP, early thymic precursor; MLL-r, MLL rearrangement; CNSL, central nervous system leukemia; CR, complete remission; HSCT, hematopoietic stem cell transplantation
